# Embryonic developmental arrest in the annual killifish *Austrolebias charrua*: A proteomic approach to diapause III

**DOI:** 10.1371/journal.pone.0251820

**Published:** 2021-06-04

**Authors:** Cora Chalar, Graciela Clivio, Jimena Montagne, Alicia Costábile, Analía Lima, Nicolás G. Papa, Nibia Berois, María José Arezo

**Affiliations:** 1 Sección Bioquímica, Facultad de Ciencias, Universidad de la República, Montevideo, Uruguay; 2 Sección Biología Celular, Facultad de Ciencias, Universidad de la República, Montevideo, Uruguay; 3 Unidad de Bioquímica y Proteómica Analíticas, Institut Pasteur Montevideo, Montevideo, Uruguay; 4 Instituto de Investigaciones Biológicas Clemente Estable, Ministerio de Educación y Cultura, Montevideo, Uruguay; 5 Laboratorio de Biología Molecular de Organismos Acuáticos, Sección Biología Celular, Facultad de Ciencias, Universidad de la República, Montevideo, Uruguay; Leibniz Institute on Aging - Fritz Lipmann Institute (FLI), GERMANY

## Abstract

Diapause is a reversible developmental arrest faced by many organisms in harsh environments. Annual killifish present this mechanism in three possible stages of development. Killifish are freshwater teleosts from Africa and America that live in ephemeral ponds, which dry up in the dry season. The juvenile and adult populations die, and the embryos remain buried in the bottom mud until the next rainy season. Thus, species survival is entirely embryo-dependent, and they are perhaps the most remarkable extremophile organisms among vertebrates. The aim of the present study was to gather information about embryonic diapauses with the use of a “shotgun” proteomics approach in diapause III and prehatching *Austrolebias charrua* embryos. Our results provide insight into the molecular mechanisms of diapause III. Data are available via ProteomeXchange with identifier PXD025196. We detected a diapause-dependent change in a large group of proteins involved in different functions, such as metabolic pathways and stress tolerance, as well as proteins related to DNA repair and epigenetic modifications. Furthermore, we observed a diapause-associated switch in cytoskeletal proteins. This first glance into global protein expression differences between prehatching and diapause III could provide clues regarding the induction/maintenance of this developmental arrest in *A*. *charrua* embryos. There appears to be no single mechanism underlying diapause and the present data expand our knowledge of the molecular basis of diapause regulation. This information will be useful for future comparative approaches among different diapauses in annual killifish and/or other organisms that experience developmental arrest.

## Introduction

During evolution, many organisms achieved the capacity to conquer extreme environments. To survive incompatible life conditions, one strategy is to enter a state of dormancy that has been reported in a range of organisms: programmed arrest during development in yeast spores, plant seeds, cysts of *Artemia*, cryptobiosis in tardigrades and rotifers, diapauses in annual killifish embryos and hibernation in mammals [1, 2].

Diapause is a reversible developmental arrest of metabolic changes and extreme stress tolerance [3]. Diapause regulation in nematodes, crustaceans, insects and fish has been reviewed. These data showed that there is no single mechanism underlying diapause, although some pathways appear to be shared [4]. In this sense, Ragland et al. [5] suggest that there may be many transcriptional strategies to produce physiologically similar dormancy responses, as there is little transcriptional similarity among dormancies across species. Nevertheless, it has been demonstrated that the transcription factor FOXO is a prime candidate for activating many physiological pathways that cause the diapause phenotype in insects and nematodes, including regulation of metabolism and the cell cycle [4].

Growing evidence suggests that inhibition of insulin-like signaling pathways is involved in cell cycle arrest at the G1 phase and plays a central role in diapause regulation in a variety of organisms; therefore, this inhibition could be a universal mechanism to control cellular physiology via metabolic modifications. Consequently, it is essential to generate more information describing how environmental signals are coupled to inner signal transduction cascades involved in the generation of diapause phenotypes [6]. Annual killifish (Cyprinodontiformes, Aplocheloidei) are freshwater teleosts from Africa and America that live in ephemeral ponds, which dry up in the dry season. The entire juvenile and adult population dies, and the embryos remain buried in the bottom mud until the next rainy season, when ponds are again flooded, embryos hatch and juveniles reach sexual maturity within a few weeks. Thus, species survival is entirely embryo-dependent [2, 7–9]. Annual killifish embryos are perhaps the most remarkable extremophile organisms among vertebrates. These embryos tolerate rather harsh environments while retaining the ability to sense and respond to critical environmental cues such as temperature, dehydration and hypoxia [10, 11].

Surviving in ephemeral environments requires the evolution of reproductive and developmental strategies that are tightly coupled to an organism’s annual life cycle. Strategies similar to the annual killifish life cycle, which is compressed in a few months, evolved in a variety of invertebrates but are uncommon in vertebrates [7, 12]. This group shares several characteristics with other widely used model fish species [9]. Nonetheless, they display unique developmental features, including the ability to undergo three reversible arrests: diapause I (DI) during the dispersed cell phase (100% epiboly), diapause II (DII) in embryos during organogenesis and diapause III (DIII) at the prehatching stage [2, 7].

DII is the most well characterized developmental arrest [13–16]. Recently, a transcriptomic analysis of DII in the African killifish *Nothobranchius furzeri* revealed that this state is actively maintained by specific regulators of chromatin and involves physiological changes that protect the organism from aging, preserving it for prolonged periods (equivalent to the half-life of adults), without consequences for the growth, fertility and life expectancy of the organism [17].

DIII occurs in the prehatching stage when the embryo has completed its development. It is characterized by a slowdown in heart rate and the cessation of development and growth. The embryo is ready to hatch if appropriate hatching cues appear [2, 7]. DIII is the least well known of the three diapauses. Scarce data indicate that embryonic metabolism decreases progressively in DIII of *A*. *limnaeus* embryos [18] compared to embryos in the same stage induced to hatch [18, 19]. A comparative transcriptomic study between DIII embryos and free-living larvae of South American *Nematolebias whitei* showed 945 differentially expressed genes. Similar transcriptional patterns were found among *N*. *whitei* and other diapausing animals in a small set of genes associated with stress resistance, circadian rhythm, and metabolism [20].

Killifish diapauses react differently to the same environmental signal (e.g., hypoxia induces and prolongs DI and II but completes DIII, [21]), which is considered an ancestral feature that has been lost several times during the evolution of nonannual killifish [22]. However, Furness et al. [23] suggested that diapause evolved by convergent evolution.

*Austrolebias* (Cyprinodontiformes: Rivulidae), a South American killifish genus, is distributed in the La Plata-Paraná-Patos-Merín and southwestern Amazon basins. The genus comprises 48 species. Most species are in the endangered category of IUCN Red List Assessments [24]. *Austrolebias charrua* (Costa y Cheffe, 2001) is distributed in lowlands that include “Bañados del Este,” an Eastern Uruguayan Biosphere Reserve and Ramsar Site [25] and southern Brazil.

Recently, studies have realized the high value of answering questions about the regulation of diapause and its possible connection with aging in vertebrates. Understanding this topic is a great challenge that goes beyond basic knowledge and can be extended in areas of biomedical concern [26, 27].

We are interested in improving knowledge of the molecular events that underlie annual killifish diapauses. To infer putative functional changes taking place, in the present study we compared *Austrolebias charrua* DIII and prehatching embryos using a shotgun label-free proteomics approach.

## Results

**Fig 1:**
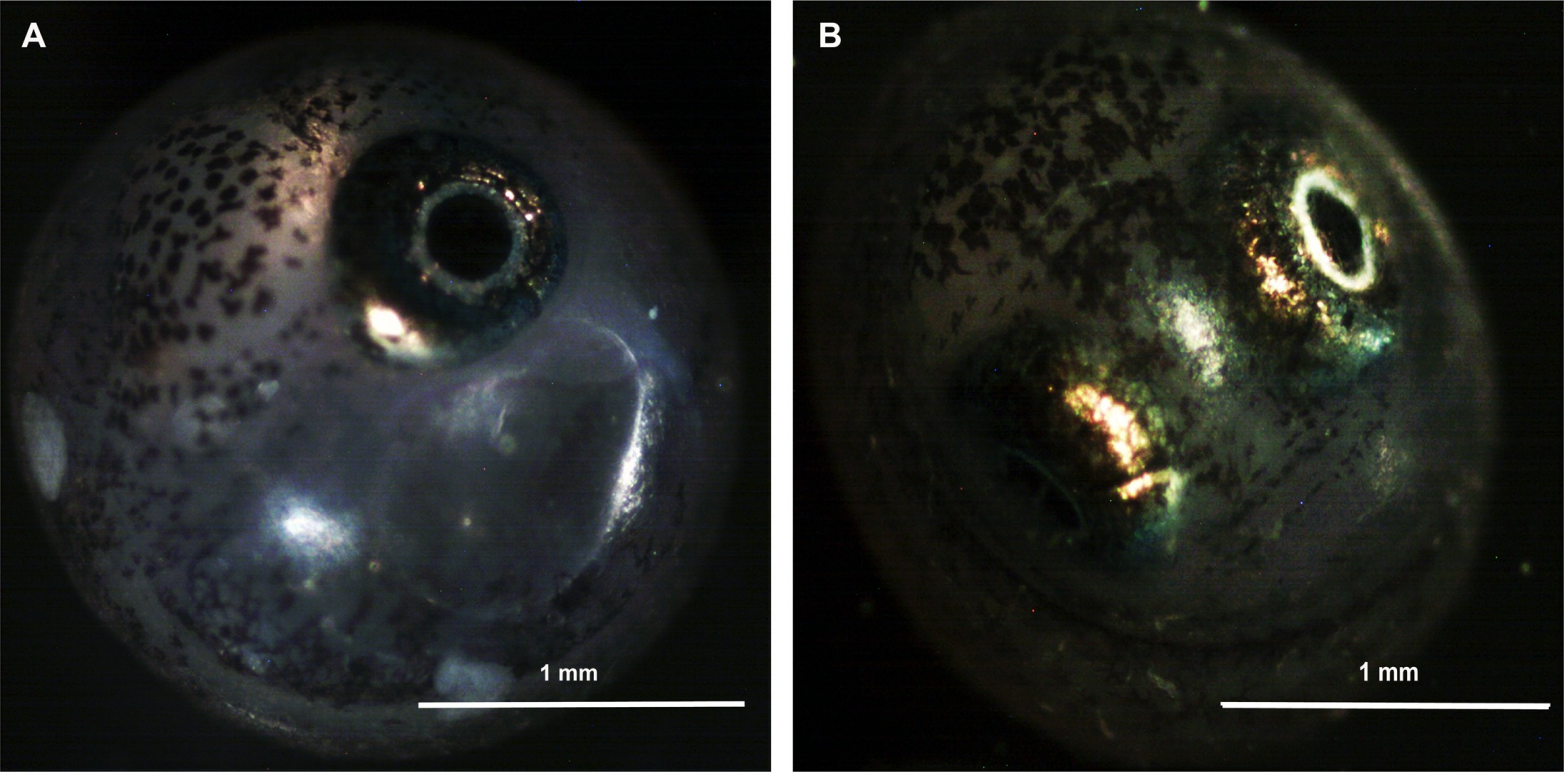
*Austrolebias charrua* embryos. A) Prehatching (PRE) (stage 41 [2]), B) diapause III (DIA) (stage 43 [2]).

The aim of the present study was to gather information about *Austrolebias charrua* diapause III (DIA) proteins with the use of 1D SDS polyacrylamide electrophoresis prefractionation combined with the use of shotgun proteomics methods. We developed a system of laboratory-grown embryos, treated them to induce DIA, and compared the proteome profiles between DIA and prehatching (PRE) using label-free LTQ (nanoHPLC-ESI-TRAP) analysis. PRE (Fig 1A) are an active developmental stage characterized by the embryo coiling about the yolk with the tip of its tail touching the rear margin of the eye. The lower jaw is capable of movement in this stage (stage 41 [2]). DIII embryos (Fig 1B) are fully developed and may hatch at any time in response to environmental cues. The heart rate slows considerably, and the embryos stop developing and growing (stage 43 [2]).

**Fig 2:**
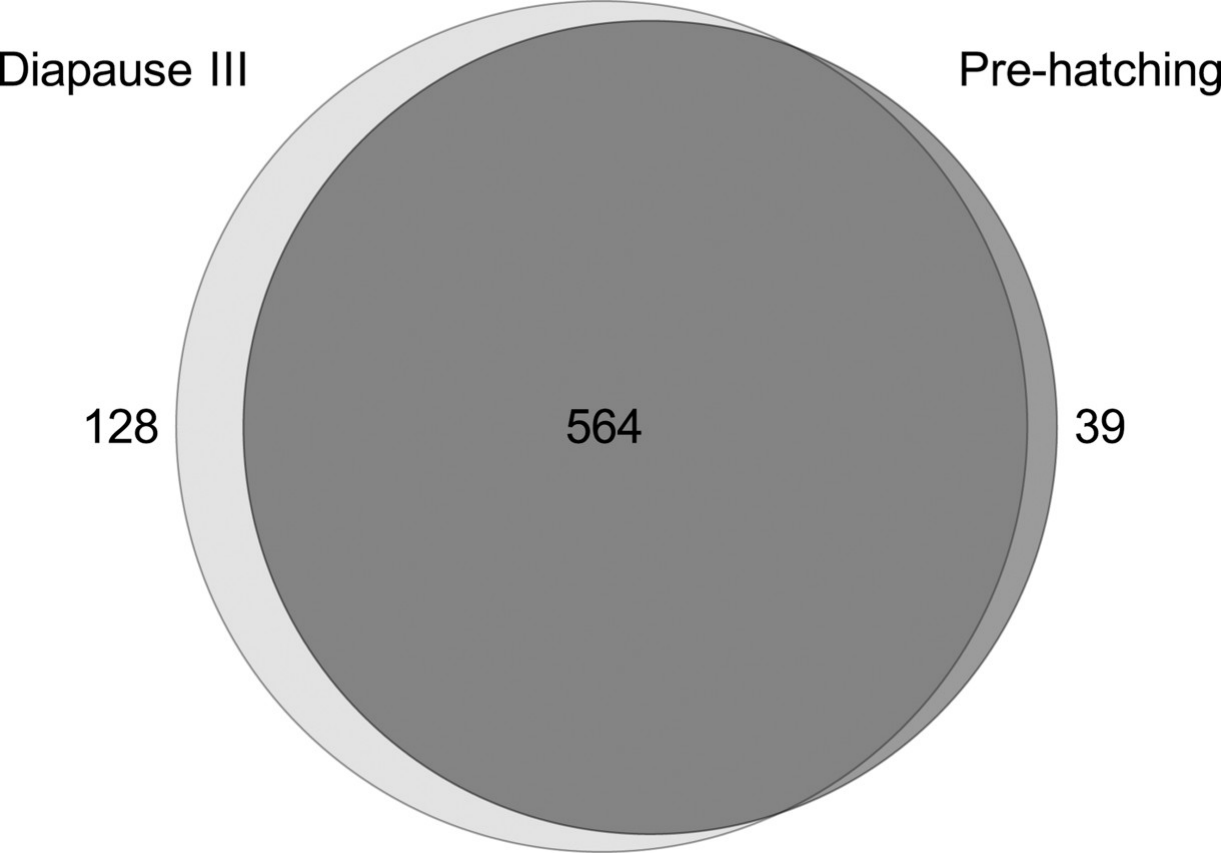
Venn diagram. Venn diagram showing proteins detected in prehatching (PRE) and diapause III (DIA). A protein was considered uniquely detected in PRE or DIA when it was identified in at least 2 replicates of one condition and was absent in the other. The number of proteins detected as uniquely present in DIA and PRE are shown light and dark gray, respectively. The overlapping section indicates the number of proteins common to both conditions.

**Fig 3:**
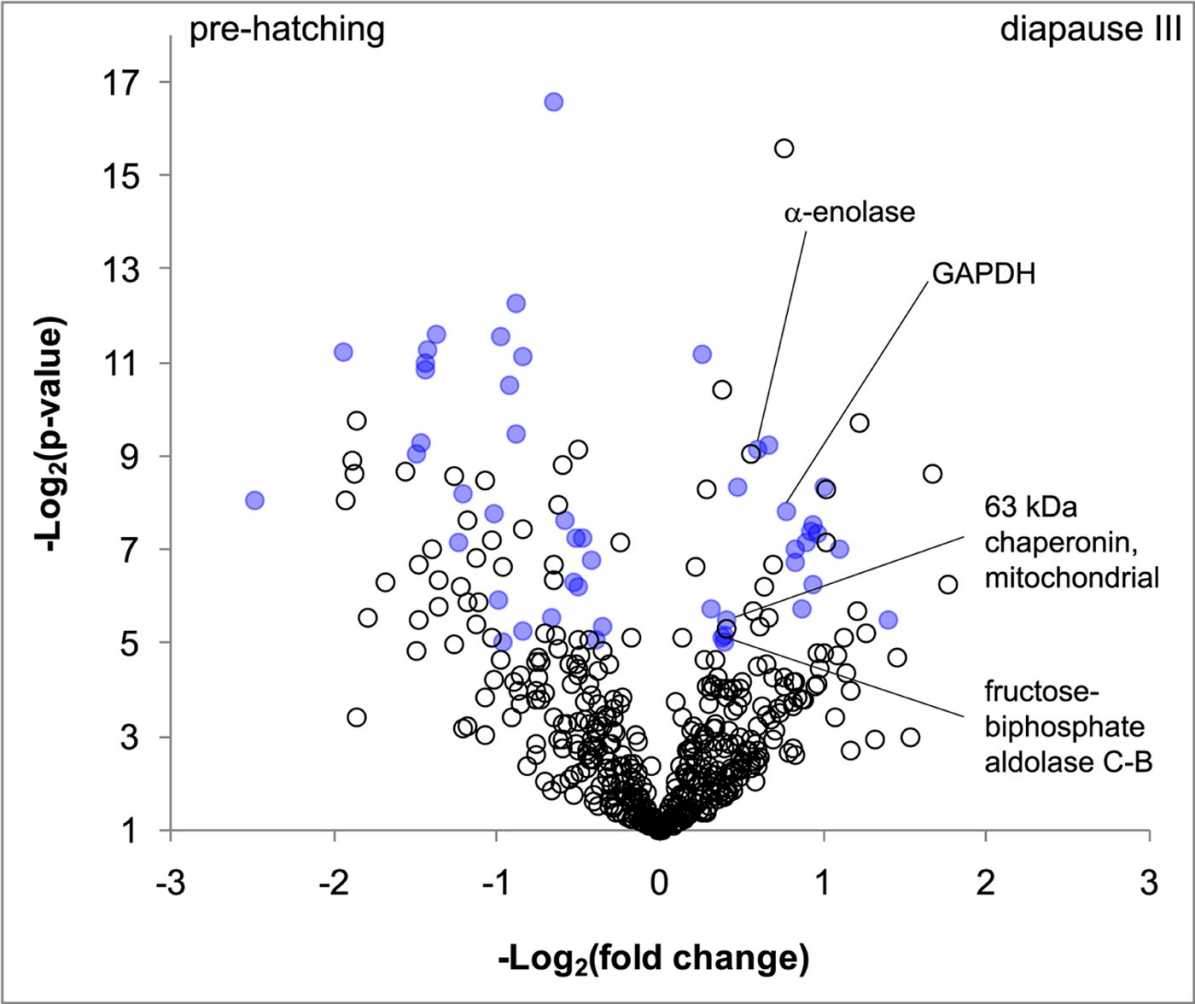
Volcano plot. Volcano plot indicating proteins present in both sample sets but with a significant difference in relative abundance according to spectrum counts. Each spot represents one protein. In the y-axis, Log2 (p-value) is indicated. In the x-axis, Log2 (fold-change) is shown (negative values indicate proteins overrepresented in prehatching (PRE) samples, and positive values show proteins overrepresented in diapause III (DIA) samples). Blue dots correspond to proteins that show a significant difference in relative abundance between conditions. Proteins of interest are indicated.

The knowledge gained from our qualitative approach is a prerequisite for a better understanding of DIII and for the identification of important proteins for this process. The overall list of proteins identified, their molecular mass, sequence coverage, number of unique peptides and the peptide sequences assigned to each protein are provided in S1 Table. In total, 654 proteins were identified in DIA and 533 in PRE. Taking into account that a protein is unique when it is present in at least two of the three replicas of one condition and in none of the replicas of the other condition, a total of 128 proteins were observed to be unique to the DIA condition, whereas 21 were differentially overrepresented. The number of unique proteins identified in the PRE condition was 39, while 28 additional proteins were identified as more abundant than in DIA (Figs 2 and 3, S2 Table).

**Fig 4:**
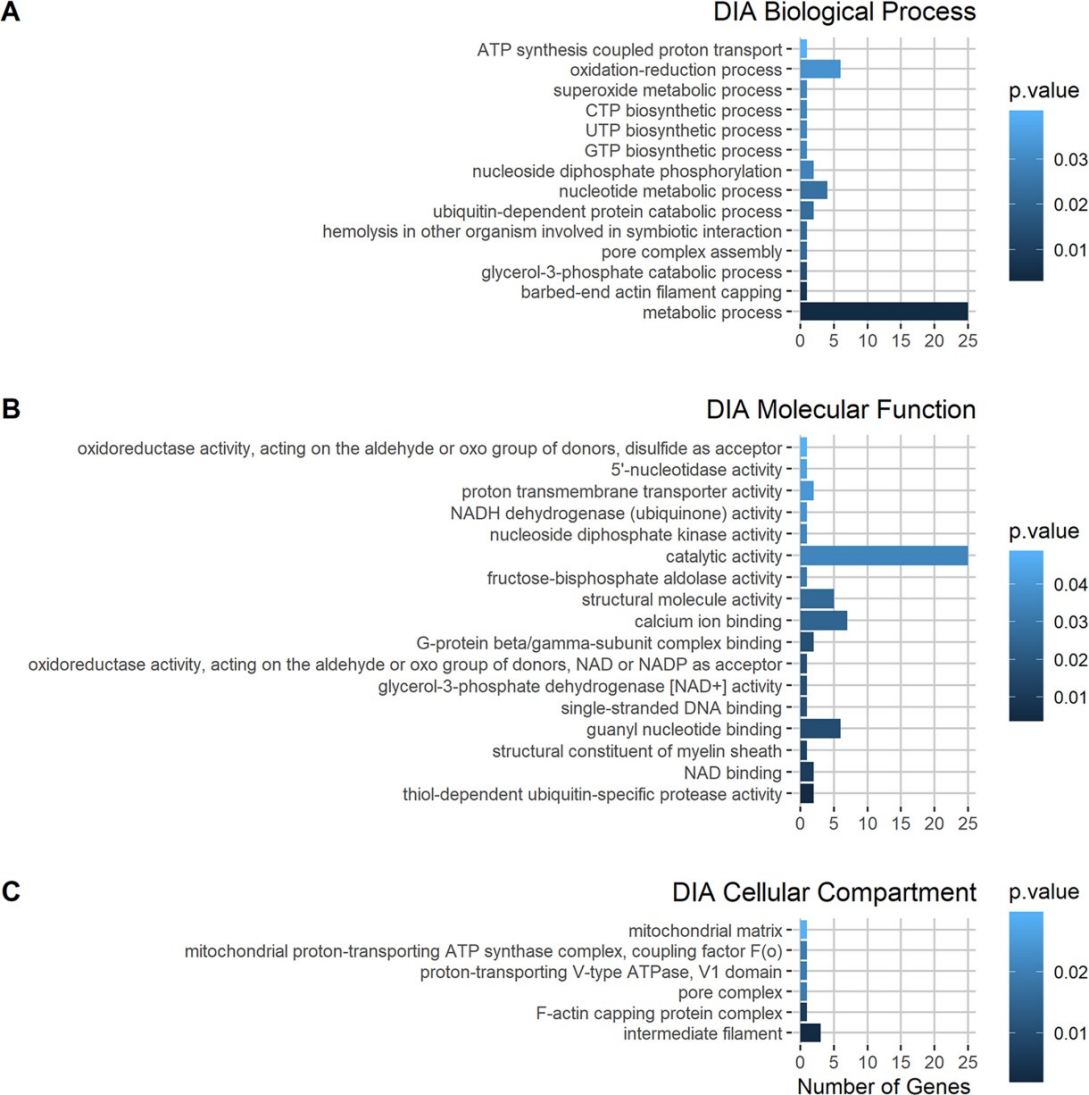
GO term enrichment of upregulated proteins in diapause III (DIA). A) Biological process GO terms, B) molecular function GO terms, and C) cellular compartment GO terms. The color of each bar corresponds to the p-value of the Fisher test (see legend of each plot). The output of the analysis and the genes associated with each GO term are shown in S3 Table.

To analyze Gene Ontology (GO) annotation, the biological functions of the identified proteins were grouped into three distinct ontologies: biological process (BP, describing sets of molecular events), molecular function (MF, describing the activities of gene products), and cellular component (CC, describing parts of a cell or its external environment). The most enriched GO terms in the BP and MF ontologies for upregulated DIA proteins (exclusive proteins plus more abundant representative proteins) were metabolic processes and catalytic activities, respectively (Fig 4), while the most enriched term in the CC ontology was intermediate filaments.

**Fig 5:**
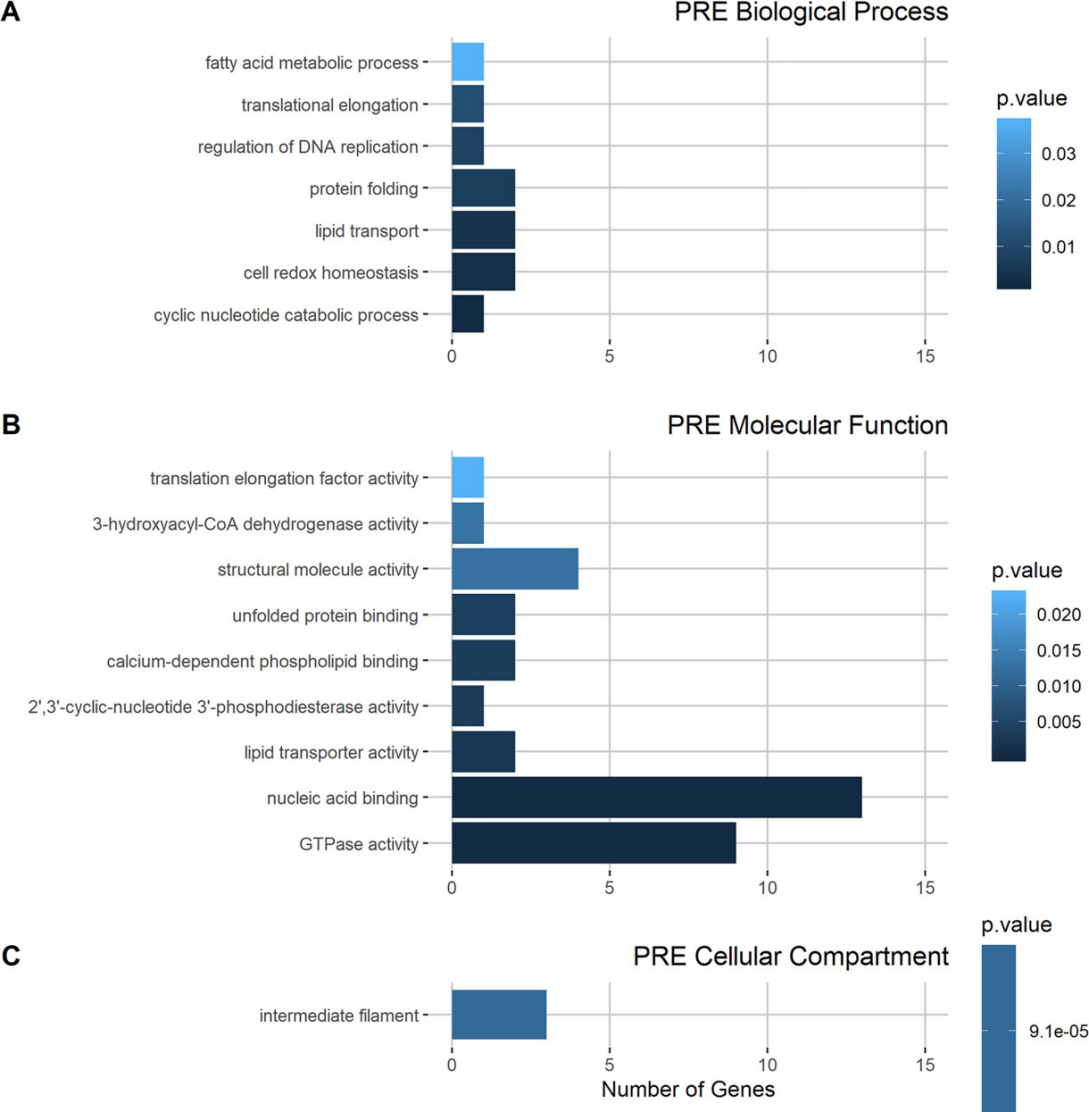
GO term enrichment of upregulated proteins in prehatching (PRE). A) Biological process GO terms, B) molecular function GO terms, and C) cellular compartment GO terms. The color of each bar corresponds to the p-value of the Fisher test (see legend of each plot). The output of the analysis and the genes associated with each GO term are shown in S3 Table.

On the other hand, the PRE proteins showed a more equitable distribution in the enrichment of the different terms in the BP ontology and differential enrichment of the terms nucleic acid binding and GTPase activity in the MF ontology. Strikingly, among the PRE proteins, the only enriched CC term was also intermediate filaments (Fig 5). However, the proteins associated with this term differed between conditions (S3 Table). Under DIA conditions, proteins associated with intermediate filaments were annotated as keratin, type I cytoskeletal 42; keratin, type I cytoskeletal 18 (K18) and vimentin. Under PRE conditions, the genes were annotated as keratin, type II cytoskeletal 8; keratin, type I cytoskeletal 17; and keratin, type I cytoskeletal 42.

**Fig 6:**
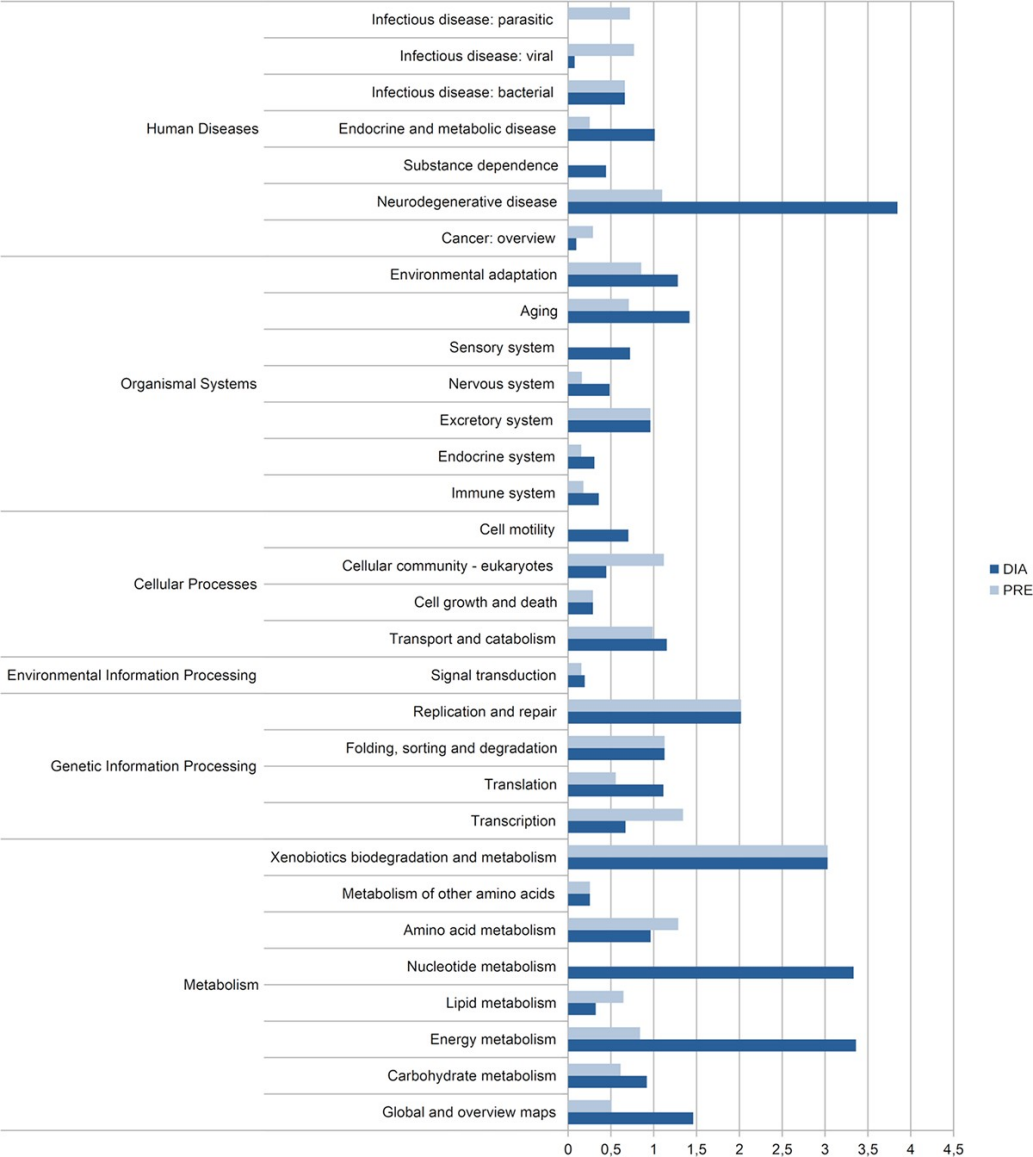
KEGG pathway analysis of upregulated proteins in prehatching (PRE) and diapause III (DIA). Bars indicate the proportion of proteins upregulated in each condition in relation to the total number of proteins of the entire proteome assigned to each pathway (PRE: light blue, DIA: blue). On the left side, the upper hierarchical category is shown. See S4 Table for details.

We carried out an analysis to identify metabolic pathways present in each condition. To assign a metabolic or regulatory pathway to each protein identified in DIA and PRE situations, the Kyoto Encyclopedia of Genes and Genomes (KEGG) pathway analysis tool was used. Comparative analysis of the assigned metabolic and regulatory pathways (Fig 6 and S4 Table) revealed that under DIA conditions, several metabolic pathways related to ATP management exhibited higher expression and were more highly represented. Likewise, some pathways, such as environmental adaptation, aging and cell motility, were also differentially expressed in DIA with respect to PRE.

It is important to note that among the proteins exclusively detected in DIA, superoxide dismutase Cu-Zn, superoxide dismutase Mn, Forkhead box protein K2, insulin-like growth factor 2-binding protein, glycerol 3-phosphate dehydrogenase, proteins involved in muscle development and function (M-protein, myozenin-1, troponin C, myomesin-3, myosin-binding protein C slow-type), periplakin, vimentin and protein NDRG2 attracted attention because they or the terms to which they are associated have been identified in other similar processes (S5 Table).

When analyzing KEGG pathway enrichment (see Table 1 and S4 Table for details), significantly enriched terms in DIA mostly consist of metabolic pathways and, to a lesser extent, cellular processes involving different types of cellular unions and endocytosis.

**Table 1:** KEGG enriched pathways in DIA and PRE.

DIA-enriched pathways			Corrected p-value
Cellular Processes	Cellular community—eukaryotes	Focal adhesion	0.0193
**Cellular Processes**	**Cellular community—eukaryotes**	**Tight junction**	**0.0456**
Cellular Processes	Transport and catabolism	Endocytosis	0.0456
Metabolism	Amino acid metabolism	Alanine, aspartate and glutamate metabolism	0.0203
Metabolism	Amino acid metabolism	Valine, leucine and isoleucine degradation	0.0310
Metabolism	Carbohydrate metabolism	Glycolysis/Gluconeogenesis	3.02E+04
Metabolism	Carbohydrate metabolism	Pentose phosphate pathway	0.0122
Metabolism	Energy metabolism	Oxidative phosphorylation	0.0123
Metabolism	Global and overview maps	Carbon metabolism	1.22E+04
Metabolism	Global and overview maps	Metabolic pathways	2.78E+05
Metabolism	Global and overview maps	Biosynthesis of amino acids	0.0042
Metabolism	Lipid metabolism	Fatty acid degradation	0.0222
Metabolism	Nucleotide metabolism	Purine metabolism	0.0052
Organismal Systems	Circulatory system	Adrenergic signaling in cardiomyocytes	0.0050
Organismal Systems	Circulatory system	Cardiac muscle contraction	0.0087
PRE-enriched pathways			Corrected p-value
Cellular Processes	Cell growth and death	Apoptosis	9.53E+06
Cellular Processes	Cellular community—eukaryotes	Gap junction	3.48E+00
**Cellular Processes**	**Cellular community—eukaryotes**	**Tight junction**	**4.43E+05**
Cellular Processes	Transport and catabolism	Phagosome	8.22E+00
Genetic Information Processing	Folding, sorting and degradation	Protein processing in endoplasmic reticulum	0.0008
Genetic Information Processing	Transcription	Spliceosome	0.0248

On the other hand, PRE-enriched pathways also involved cellular processes, including other cellular union types, apoptosis and phagosomes, as well as genetic information processes, such as spliceosome and protein processing in the endoplasmic reticulum. Strikingly, the cellular process tight junction was enriched in both conditions. A closer inspection of proteins associated with this term revealed that upregulated proteins in each condition led to different outcomes (S4 Table). In DIA, proteins included Arp2/3, PKA, Myosin II, Rab8 and Rap1a, while in PRE, proteins included PP2A, PCNA and several tubulins. PP2A (serine/threonine-protein phosphatase 2A) inhibits the cell polarity process through the aPK2 protein and inhibits actin and tight junction assembly through inhibition of occludin.

These results indicate that the two conditions analyzed have very different upregulated cellular functions.

**Fig 7:**
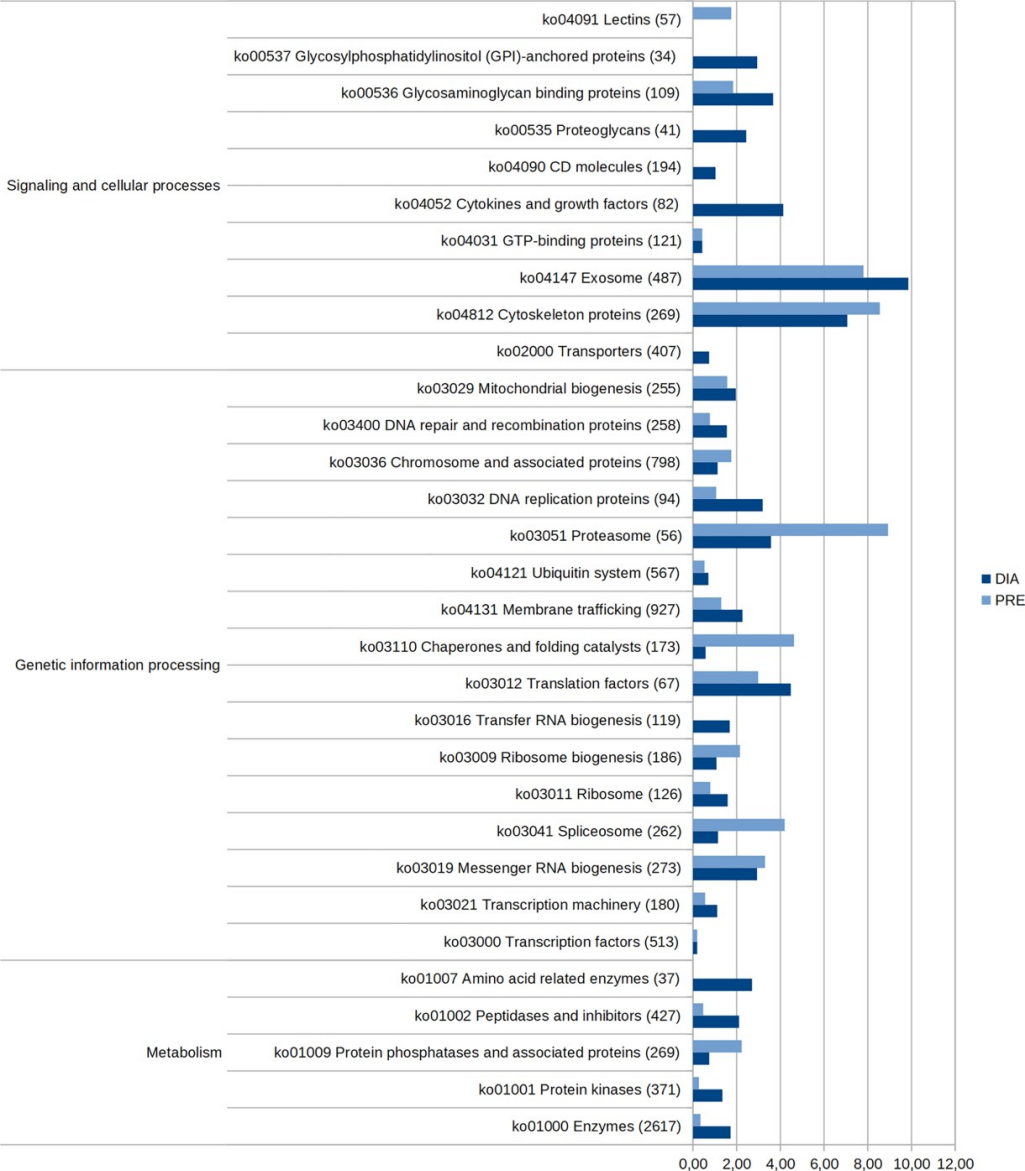
Brite classification of upregulated proteins in prehatching (PRE) and diapause III (DIA). Bars indicate the proportion of proteins upregulated in each condition in relation to the total number of proteins of the entire proteome assigned to each category (PRE: light blue, DIA: blue). On the left side, the upper hierarchical category is shown.

Proteins exclusive or upregulated in each condition were classified according to KEGG Brite hierarchical classifications of biological entities. Fig 7 shows DIA and PRE proteins in each category. In PRE, enriched categories include proteasome and chaperones, as well as spliceosome proteins and phosphatases. On the other hand, several categories seem to be exclusive to the DIA condition, such as tRNA biogenesis, amino acid-related enzymes, transporters, cytokines, growth factors, CD molecules, and GPI-anchored proteins. In addition, enzymes, kinases, replication proteins and peptidases were more enriched in DIA than in PRE.

## Discussion

Diapause is a phenomenon of wide interest that occurs in a large number of organisms from prokaryotes to mammals [1]. Nevertheless, proteomic data about diapause are scarce, particularly in nonmodel organisms presenting this kind of dormancy during embryonic development. The goal of this study is to shed light on the mechanisms involved in diapause III in *A*. *charrua*, a South American annual killifish species, by quantitative proteomics. It is essential to consider that protein levels may not fully reflect the functional levels of a specific protein within a pathway. On the other hand, low or high abundance or detection in one state and absence (below the detection level) in another group provides insight into putative functional directions [28]. Additionally, the experimental design developed in this study does not allow for the discrimination of changes in protein abundance as a result of dormancy in diapause III and the effects of environmental dehydration. Nevertheless, it is highly conceivable that the two processes often co-occur in nature. According to the only two studies about field data available, researchers found *Millerichthys robustus* and *Nothobranchius* embryos arrested in DIII close to the end of the dry season before flood [29, 30].

Considering the restrictions mentioned above, proteome profiles were analyzed in an attempt to supply an interpretation of the roles of the identified proteins in support of dormancy.

Podrabsky et al. [11] demonstrated that post diapause II embryos exhibit stress resistance after diapause exit for the first 4–6 days of post-DII development resumption. Based on these findings, we opted to use embryos that did not enter DII and selected the prehatching stage (ST 41 [2]) to compare the differential protein expression profile against DIII (ST 43 [2]). Nevertheless, it is important to take into account that many prehatching embryos at stage 41 could be prepared to enter diapause since this developmental arrest is considered obligate for most annual killifish studied [7].

It is essential to consider that although diapause implicates an arrest of development, the arrested state is not static. Diapause usually involves considerable physiological changes that help to prepare the organism to survive upcoming environmental challenges [31]. Recently, in a transcriptomic approach, Hu et al. [17] demonstrated that diapause is a highly active state with significant changes in gene expression during both induction and maintenance of diapause. The authors found that genes whose expression is most strongly induced during diapause are regulators of chromatin of the Polycomb group of transcriptional repressors and genes involved in muscle development and maintenance. In DIII *A*. *charrua* embryos, proteins of the Polycomb group were not found. It is possible that these regulatory genes were expressed in low quantities and were difficult to identify by the method employed in this study. However, the expression of proteins involved in muscle development and function was detected in DIA, suggesting an important role for muscle maintenance during this diapause, as indicated by Hu et al. [17] for *N*. *furzeri* DII embryos. These authors also detected ribosomal proteins and hydrolases implicated in nucleotide and amino acid metabolism. Furthermore, these types of proteins were found in DIA *A*. *charrua* embryos.

### Stress tolerance and proteins involved in damage prevention

Our results suggest that diapause embryos could respond by significantly altering their metabolic profile due to the dehydration stress imposed by the environment. Embryos subjected to dehydration also have greater oxygen availability, and in this sense, the dehydration stress would increase reactive oxygen species (ROS) formation that can lead to lipid peroxidation, denaturation of proteins, and DNA damage [32]. It is known that organisms regulate their defenses against oxidative stress by increasing the production of antioxidant enzymes when dehydrated [33].

Taking this into account, the embryo should trigger a response consecutive to changes in environmental conditions, which could involve biosynthesis of a number of new molecules, particularly proteins involved in the scavenging of ROS. Among the proteins known for their protective function against oxidative damage, the most notable change found in DIA concerns two superoxide dismutases (SOD), Cu/Zn SOD and Mn SOD. These are metalloenzymes with a primary role of catalyzing the conversion of superoxide radicals to molecular oxygen and hydrogen peroxide, which are then enzymatically converted by catalase into molecular oxygen and H2O. Cu/Zn SOD is usually the most abundant SOD and can be found in the cytosol and in the extracellular space, while Mn SOD is a mitochondrial matrix enzyme that scavenges oxygen radicals produced by the oxidation–reduction and electron transport reactions that occur in the mitochondria [34]. Although catalase was present in our study, no change was detected under the conditions tested.

Wagner et al. [35] showed that tolerance to H2O2 reaches a peak during *Austrofundulus limnaeus* DII, while DIII embryos exhibit the lowest tolerance of all stages studied. However, these authors saw that protection against oxidative damage relies on small-molecule antioxidants during early development and on enzymatic systems towards the end of embryonic development, although most enzymatic antioxidant activity did not seem to depend on SOD [35]. Nevertheless, strategic antioxidant protection associated with increased SOD expression was described for other diapausing organisms [36]. Interestingly, it has been suggested that FoxO plays a conserved role in setting diapauses [37].

The targets of the FoxO transcription factor include genes that encode antioxidant proteins whose function is to eliminate ROS. Among these targets are human Mn-SOD and murine Cu/Zn-SOD. Catalase is also regulated by FoxO [38]. Although FoxO was not among the proteins identified in *A*. *charrua*, as a transcription factor its expression level can be low, and it is worth looking specifically to see if it also exhibits differential expression during DIA.

It is also striking that NAD/NADP-dependent betaine aldehyde dehydrogenase (ALDH) was expressed exclusively in DIA. ALDH enzymes metabolize aldehydes derived from lipid peroxidation during oxidative stress, many of which are extremely reactive and toxic. Upregulation of ALDHs is a practically universal response against a variety of environmental stressors, such as dehydration and ultraviolet radiation. In *C*. *elegans* during the dauer life stage, the signaling pathways that modulate antioxidant skills induce the expression of ALDHs [39]. In addition, in plants, the toxic aldehyde intermediate betaine aldehyde is transformed into glycine betaine by the same ALDH isoform that is exclusively expressed in DIA. Glycine betaine is an osmolyte that protects plants from dehydration and other unfavorable conditions, such salinity, cold and oxidative stress [40].

The proteins contained in the yolk are the source of amino acids that can have a direct role as building blocks for new protein synthesis or can sustain the generation of energy in the embryo. Among exclusive DIA proteins, the one with the highest representation was glutamate dehydrogenase (GDH), an enzyme that catalyzes oxidative deamination of the amino acid glutamate, generating α-ketoglutarate and ammonia while reducing NAD+ or NADP+. The obtained α-ketoglutarate is metabolized by the Krebs cycle, leading to the synthesis of ATP. In addition, GDH function is linked to the maintenance of cellular redox homeostasis. GDH controls the intracellular levels of its product α- ketoglutarate and controls the next TCA metabolite, fumarate, which binds to and activates a reactive oxygen species that can be scavenged by the enzyme glutathione peroxidase [41]. Although this may be of great importance for diapause embryos since they must overcome stressors, another factor must be considered. The alpha-ketoglutarate obtained by GDH could produce2-hydroxyglutarate in a reaction catalyzed by 3-phosphoglycerate dehydrogenase (PHGDH) or by the action of malate dehydrogenase [42, 43] Of the two enzymes, the only one present in the current work was the malate dehydrogenase (but without reaching the required levels to be considered differentially expressed). Interestingly, a metabolomic analysis carried out in *Austrofundulus limnaeus* embryos suffering from dehydration stress during their diapause detected the metabolite 2-hydroxyglutarate [44]. The 2-hydroxyglutarate competitively inhibits α-ketoglutarate-dependent dioxygenases, and among its targets are prolyl hydroxylase domain (PHD) proteins, which are critical elements in stabilizing the hypoxia inducible factor HIF1α and hence the regulation of the transcription factor HIF-1. This factor plays a prominent role in the metabolic adjustments that drive cellular adaptation to low oxygen availability [45]. However, since DIII embryos have abundant availability of oxygen, the responsibility of arranging metabolic changes would not fall on HIF-1 coordination. Similarly, if there were changes in the epigenetic scenery of *A*. *charrua* DIII as suggested by changes that we will discuss later, they most likely would not adhere to the inhibition of DNA demethylases and histone demethylases by 2-hydroxyglutarate.

Heat shock proteins (HSPs) are damage prevention proteins that rapidly change their expression patterns in response to environmental shock. It is well established that HSP expression can change on several diapauses; however, an increase does not always occur. During diapause of insects, HSPs may increase, decrease, or remain unchanged [46]. For example, it is argued that HSPs would not play any role during *Bombyx mori* diapause because its expression does not change between diapause and nondiapause eggs [47, 48].

In *Sarcophaga crassipalpis*, HSP90 expression is high in nondiapausing and early diapause-destined individuals but drops significantly thereafter [49], and *Nasonia* presents this same expression pattern [48]. Meanwhile, our results indicate that HSPs were present in PRE and DIA, with only the 63 kDa chaperonin overrepresented in DIA. HSP90 and cognate HSP70 and HSP71 were overrepresented in PRE, while the protein that interacts with Hsc70 was exclusive to this stage. This scenario could be a sign that changes in HSPs occur prior to rather than in reaction to diapause, suggesting that HSPs would be ready to protect proteins from irreversible denaturation imposed by the stress associated with diapause. A similar method was previously proposed by King and MacRae [50]. The only available "omic" study of DIII is a transcriptomic approach in the annual killifish *Nematolebias whitei* [20]. This study showed high differential expression of HSP cognate 71 kDa protein-like between DIA and free-swimming larvae. In our study, this protein appeared to be overrepresented in PRE, but given that the present proteomic study was performed in a different species, we do not know what happens to the expression of messenger RNA in DIA embryos of *A*. *charrua*. In addition, we cannot ignore the fact that these results reflect what occurs in a time window; therefore, kinetic studies should be carried out to obtain a complete picture of *A*. *charrua* embryo HSPs expression.

Although increased expression of proteins with alpha-crystallin domains with chaperone or heat-shock functions during dormancy has been identified in different organisms, particularly invertebrates [46, 51], the six diapause increased proteins identified as crystalline in the present work were from the beta and gamma families. This suggests that the observed overrepresentation could be due to the lens growth of the embryo between the prehatching stage (ST 41) and DIII (ST 43).

### Energy metabolism

It is usually assumed that during diapauses, metabolism is depressed; however, our results suggest metabolic changes that appear to indicate energy metabolism enhancement compared to the prehatching stage. DIA proteome profiling would indicate increased function of glycolysis that would not generate biomass if the diapause embryo does not grow or proliferate [2, 7, 18]. (we must also note that the cell cycle could be arrested by DIA periplakin and vimentin exclusivity as we will discuss later).

Glycolysis is the front door to obtain metabolic energy from carbohydrates, while gluconeogenesis works in the opposite direction, although it shares several steps with the glycolytic process. However, the glycolysis rate-limiting enzyme hexokinase exclusively found in DIA suggests that the glycolysis pathway was increased under diapausing conditions, utilizing glucose derived from the yolk’s glycogen [52]. Interestingly, concerning the DIA hexoquinase isotype (hexoquinase I), different experimental approaches concluded that the enzyme is bound to actively phosphorylating mitochondria facilitating the access to intramitochondrial ATP as substrate. This would further coordinate the first glycolysis reaction in the cytosol with the final oxidative stages of glucose metabolism in the mitochondria, thus ensuring an overall rate of glucose metabolism in line with cellular energy demands [53]. Furthermore, binding of hexoquinase I to mitochondria prevents Bax-induced cytochrome c release and therefore this binding inhibits apoptosis [54–56]. The hexokinase I-mitochondria interaction controlling both bioenergetics and cell death, aspects that would be relevant in diapause III, undoubtedly presents great interest and deserves to be explored. In addition, several enzymes involved in glycolytic metabolism were significantly more abundant in DIA, such as phosphoglucomutase, glyceraldehyde 3-phosphate dehydrogenase (GAPDH), aldolase and enolase, some of which are known to be upregulated during insect diapauses [48]. Similarly, Hussein et al [57] showed that glycolysis was the most significantly upregulated metabolic pathway from the preimplantation to the diapause stage based on RNA-seq analysis conducted on mouse blastocysts. Since mouse blastocyst diapause is a cell cycle arrest and growth dormancy stage, these authors pose an open question: why does diapause require such a high rate of glycolysis? It is important to bear in mind that glycolysis is among the biochemical changes created by the stress response that accompanies diapauses. In some cases, its activation is oriented towards glycerol biosynthesis, as seen in overwintering insects and in hibernating black bears [58, 59]. Under normal conditions, glycerol is mainly used to make triacylglycerols and phospholipids. However, some organisms exposed to cold environments use glycerol as a cryoprotectant for their macromolecules, and others accumulate glycerol during diapause and use it as an energy resource when diapause ends [60]. Glycerol-3-phosphate dehydrogenase (GDH) catalyzes the redox conversion of dihydroxyacetone phosphate to glycerol-3-phosphate, which can be used to generate glycerol. In *A*. *charrua*, the GDH protein was found specifically, in DIA. If glycerol synthesis would be increased, there could be fewer precursors available for glycolysis. However, GAPDH was also increased in DIA. Hence, it could be necessary for the embryo to alternate between these options, emphasizing GDH or GAPDH. Based on their own results in diapausing *Nasonia* and on older data from *Bombyx mori* diapause, Wolschin and Gadau [48] proposed a pathway in which switching between glycerol synthesis and glycolysis would limit glycerol gain to a restricted time window to ensure sufficient glycolysis. Recently, it was found that GDH is expressed specifically in the dormant brain tumor stem cell (BTSC) population. In a metabolomic assay, it was shown that the most notably enriched pathway in these cells is the taurine and hypotaurine pathway, which is significantly downregulated in GDH KO BTSCs. This pathway is essential for the cell stress response, as taurine is responsible for protection during osmotic stress and hypotaurine is protective during oxidative stress [61]. In this sense, glycerol-3-phosphate dehydrogenase is essential for *Saccharomyces cerevisiae* growth under osmotic stress, and significant induction of this enzyme was observed under abiotic stresses in maize [62, 63]. The increased level of DIA pyruvate dehydrogenase would provide acetyl-CoA to the Krebs cycle. Likewise, we found that the DIA citric acid cycle (TCA) could exhibit greater speed given the higher succinyl-CoA synthetase level. This enzyme functions in the TCA, coupling the hydrolysis of succinyl-CoA to the synthesis of either ATP or GTP, and thus represents the only substrate-level phosphorylation step in the cycle. In diapausing embryos, TCA could also gain acetyl-CoA derived from the enrichment of enoyl-CoA hydratase, which is essential to metabolize fatty acids via beta oxidation. The metabolic needs of lipids during killifish DIII are also covered by the yolk, which would fulfill a role similar to that of the insect fat deposits. These are the most important reserves used by insects to satisfy their energy demand during diapause, and it has been proposed that interactions between nutrient storage and metabolism can influence this diapause by utilizing signaling pathways [64]. Interestingly, our results showed that a member of the cyclic AMP signaling pathway, cAMP-dependent protein kinase, was increased in DIA. PKA-mediated protein phosphorylation is considered a major factor in the mobilization of triglycerides in insects, but this is an evolutionarily conserved mechanism that is also present in vertebrates [65]. Furthermore, PKA pathway-mediated fat mobilization is required for other stress situations in *Caenorhabditis elegans* [66]. In this transduction pathway, arrestins play an important role in regulating the function of G protein-coupled receptors, including receptor desensitization and internalization. Arrestin-3 (also called c) is phosphorylated by casein kinase II, and it is the phosphorylation state of arrestin-3 that promotes internalization of the phosphorylated receptor [67]. Importantly, while arrestin was expressed in DIA, casein kinase II was only expressed in PRE. This profile, which suggests that arrestin would remain inoperative during diapause, deserves further investigation. The role played by the DIA exclusive guanine nucleotide-binding protein (i) subunit alpha-1, which can act as an inhibitor of adenylate cyclase, thus modulating the signal, or in another pathway as a stimulator of K+ channels, also deserves attention [68]. For mitochondrial energy production, the reducing equivalents generated by the TCA cycle are transferred to the electron transport chain (ETC). Subunits of complex I, the core protein of complex III and electron transfer flavoprotein (ETF), were found exclusively in DIA. While ETF transfers electrons to ETC via ubiquinone, both complexes are proton pumps that collaborate in gradient formation and drive the synthesis of ATP by ATP synthase.

Importantly, mitochondrial ATP synthase subunit g, which enables the transfer of protons from one side of a membrane to the other, was also exclusive to DIA, suggesting that ATP synthesis might be increased.

Emphasizing the lack of confirmatory functional studies, previous proteomic data suggest that in diapause would be increased the glycolysis, leading to an enhance in aerobic metabolism, probably facilitating dehydration stress tolerance and the selective synthesis of protective molecules. In agreement with an increase in protein synthesis, high levels of molecules involved in the translational machinery were found only in diapausing embryos: ribosomal proteins, eukaryotic translation initiation factors, elongation factor 1-alpha 1 and nascent polypeptide-associated complex subunit beta. These proteins are crucial elements in translation and protein synthesis regulation, and its increase could be indicating that both processes would potentially be more active in DIA than in PRE embryos although these must be validated by biochemical approaches.

Additionally, the enrichment of nucleotide metabolism attracts our attention considering that the diapausing embryo could not proliferate as in the case of *Austrofundulus limnaeus* embryos in diapause II [13]. As it was already mentioned above, we must also note that the cell cycle could be arrested by DIA periplakin and vimentin exclusivity as we will discuss later.

The increase of nucleotide metabolism is mainly linked to the increase in proteins of the purine synthesis pathway. Related to this rise could be the striking fact that several proteins involved in GDP/GTP exchange reactions were exclusively expressed in *A*. *charrua* DIA (Rho GDP-dissociation inhibitor 1, Ras-related protein Rap-1, Ran-specific GTPase-activating protein, Ras-related protein Rab-5A, guanine nucleotide binding protein G(i) subunit alpha-1, Rab GDP dissociation inhibitor beta, guanine nucleotide-binding protein G(k) subunit alpha, and Ras-related protein Rab-8A).

According to this it was found that the induction of purine synthesis enzymes is associated with environmental changes in some diapause insects [69].

### Cytoskeleton and cell signaling proteins

Intermediate filaments are structures that provide mechanical and stress coping resilience to cells. A universal response to stress is the reorganization of the intermediate filament network. This event was most comprehensively studied in the context of keratins [70]. It is interesting to note that K18 keratins were found in DIA and K8 in PRE in our analysis. Epithelial tissues contain different keratins expressed in a tissue-specific manner. Keratin pair 8/18 is found in the intestine, liver, and pancreas [71]. This pair have a major role in the mechanical stability and integrity of cells but also executes functions in different cellular processes, including protection from apoptosis [72, 73], cellular stress response [74, 75] and cell signaling [76, 77].

In this context, it is likely that K18, which was found exclusively in DIA embryos, would be involved in protection from apoptosis and cellular stress responses during DIII in *A*. *charrua*. Moreover, it has been reported that K18 phosphorylation at Ser34 in the head domain promotes its solubility and binding to adaptor protein 14-3-3. Overexpression of K18-S34A, K8 and K18 filaments in mouse pancreatic acinar cells induces them to retract from the basal nuclear region and become apically concentrated, and nuclear retention of adaptor protein 14-3-3 leads to partial mitotic arrest in the liver [78]. It has been shown that the 14-3-3ζ (zeta subunit) protein functions as a central node to promote chemoresistance pathways in cancer [79]. The 14-3-3ζ (zeta subunit) was identified as exclusively expressed in *A*. *charrua* DIII embryos. Taken together, these data suggest that 14-3-3ζcould play a role in mitotic arrest and cell survival maintenance during DIII in *A*. *charrua* embryos.

Another exclusive cytoskeletal protein found under DIA conditions was vimentin. Vimentin is a main component of the cytoskeleton networks, although accumulating evidence places vimentin at the cell surface or in the bloodstream, either soluble or as a vesicle-transported protein, suggesting it has important roles in the extracellular environment. Cell activation, apoptosis, senescence, inflammation and stress promote vimentin secretion [80, 81]. This protein acts as an integrator of cellular mechanical functions, including cell migration, adhesion and division [82]. It is also involved in organelle positioning, homeostasis and aggresome formation and protects the nucleus during mechanical stress. Moreover, it plays important roles in cell signaling, lipid metabolism, and gene expression [83]. Recently, it was suggested that vimentin acts as an organizer of proteins that regulate the balance of protein synthesis, refolding, and degradation. Morrow et al. [84] demonstrated the role of vimentin as a critical regulator of proteostasis in neural stem cells (NSCs) and the role of aggresomes in NSCs, which clear proteins during quiescent activation. It is possible that vimentin may play a role in safeguarding proteins aligned with HSPs during DIII in *A*. *charrua* embryos.

Periplakin, also identified as an exclusive DIA protein, is a cytolinker from the plakin family [85]. This protein is an interaction partner of protein kinase B (PKB or Akt) and localizes to the cell membrane, nucleus, mitochondria and the intermediate filament network through direct binding to vimentin. Periplakin localization is in part influenced by the presence of vimentin, suggesting that periplakin may act as a scaffold and as a localization signal for PKB (Akt) signaling [86]. Interestingly, as stated by Heuvel [86], the interaction of periplakin with PKB (Akt) and vimentin can lead to the inhibition of PKB (Akt)-mediated FOXO phosphorylation and modulation of transcriptional activity. Sequestering PKB (Akt) in the cytosol through binding periplakin/vimentin enhances FOXO transcriptional activity and cell cycle arrest [86]. As stated by Hand et al [4], the transcription factor FOXO is a prime candidate for activating the diapause phenotype in insects and nematodes. It is possible that during DIA, the cell cycle could be arrested by this mechanism, as periplakin and vimentin were found to be exclusive DIA proteins.

A member of the Forkhead box (FOX) family of transcription factors, the Forkhead box protein K2, was also identified as an exclusive protein under DIA conditions. Members of this family are known to regulate a wide range of biological processes, including metabolism, cell cycle progression, proliferation, survival, differentiation and apoptosis [87]. It has been shown that together with Forkhead box protein K1/K2 acts as negative regulators of autophagy in skeletal muscle cells in response to starvation. Interestingly, Forkhead box protein K2 enters the nucleus, binds the promoters of autophagy genes and represses their expression, preventing proteolysis of skeletal muscle proteins [88]. Forkhead box protein K1 and Forkhead610 box protein K2 were identified using a proteomics approach as targets of insulin action [89]. These transcription factors, in contrast to FoxO1, are translocated from the cytoplasm to the nucleus after insulin stimulation, a pattern that is reciprocal to that of FoxO1 after insulin stimulation. Forkhead box protein K1 and Forkhead box protein K2 are critical components in IR and IGF1R-mediated signal transduction, controlling cell proliferation and metabolism [90]. These factors are present in the cytoplasm under basal conditions, and inside the nucleus, they can both activate and repress gene expression [90].

Forkhead box protein K1 and Forkhead box protein K2 also positively regulates WNT/beta-catenin signaling by translocating DVL proteins into the nucleus [91]. Interestingly, the N-Myc downstream-regulated gene 2 (NDRG2) protein was found in DIA *A*. *charrua* embryos. This protein contributes to the regulation of cell proliferation, apoptosis and metastasis by regulating various signaling pathways, including the WNT/β-catenin, mitogen-activated protein kinase, transforming growth factor β and Akt signaling pathways [92–94]. Insulin-like signaling pathways are considered to play a central role in diapause regulation in a variety of lineages [6]. Insulin-like growth factor 2 mRNA-binding protein (IGFBP2), a member of this pathway, was also found to be an exclusive DIA protein in embryos. This suggests that this pathway could also be involved in the generation of this diapause phenotype, representing a universal mechanism to control cellular physiology via metabolic modifications, as stated by Podrabsky and Hand [6]. IGFBP2 generally inhibits IGF activity, mainly IGF2 [95]. However, it is important to mention that IGFBP2 also shows IGF-independent activity, playing a role in the regulation of energy status and metabolism [96].

### DNA repair protein

Remarkably, in our analysis a deoxyribodipyrimidine photolyase was exclusively found in DIA embryos. These enzymes are involved in the repair of UV radiation induced DNA damage. They catalyze the light-dependent reversal of cyclobutane pyrimidine dimers, which are formed between adjacent bases on the same DNA strand upon exposure to ultraviolet radiation [97]. Wagner and Podrabsky [98] reported that embryos at the disperse blastomere stage (the stage at which DI can occur) and DII embryos of *A*. *limnaeus* possess an impressive tolerance to UV‐induced DNA damage. Their repair capacity appears to be strongly dependent on photolyase activity to reverse DNA lesions, suggesting that either embryos at these stages constitutively express a photolyase or that the expression of this enzyme is rapidly inducible. Taken together, these results suggest that the repair capacity of embryos during diapauses appears to be strongly dependent on the photo repair pathway.

### Protein related to epigenetic changes

Another protein found exclusively in DIA is S-methylmethionine homocysteine S-methyltransferase. This enzyme is involved in methionine metabolism, catalyzing the reaction between homocysteine and S-methylmethionine, to produce two molecules of methionine. Methionine, or its metabolites participate in protein synthesis but also in the transmethylation pathway, where methionine is converted to S-adenosyl-L-methionine (SAM), a compound that participates in most biological methylation reactions. SAM, which is involved in chromatin methylation events, is critical for the maintenance and transmission of epigenetic information [99]. Indeed, in a recent transcriptomic study conducted in the annual killifish *Nothobranchius furzeri* embryonic diapause, Hu et al [17] found upregulated genes that are implicated in chromatin regulation through their abilities to methylate histone H3. There is also evidence indicating that epigenetic silencing plays a regulatory role in insect dormancy and growth arrest in response to the environment in plants [100, 101]. Although we did not obtain differentially abundant proteins directly linked to chromatin modification, we found that the presence of S-methylmethionine homocysteine S-methyltransferase in *A*. *charrua* DIA embryos could generate methylation events during diapause. This is a topic that deserves further investigation.

### Environmental sensing

KEGG pathway analysis of overrepresented proteins in PRE and DIA (Fig 6) showed that the proportion of proteins in the “environmental adaptation” and “sensory system” categories increased in DIA conditions in relation to PRE.

One possible explanation is related to the fact that embryos in diapause could be able to tolerate rather harsh environments while retaining the ability to sense and respond to critical environmental cues [11]. Likewise, although maternal influences may program the embryo for one trajectory, environmental cues experienced by the embryo can override those maternal influences [102, 103]. Moreover, it is possible that embryos in DIA could be coupled in an inactive state, ensuring a quick response to react to the output signal of diapause and eventual hatching. It is important to consider that DIII embryos, unlike DII embryos, are ready to hatch, swim, and feed.

## Conclusions

The present study offers a first glance into global protein differences between prehatching and diapause III that could be involved in the induction / maintenance of this developmental arrest in *A*. *charrua* embryos. We found evidence of protein changes in several metabolic pathways and abundance changes in important proteins involved in stress tolerance, damage prevention, energy metabolism, cytoskeleton, cell signaling pathways, DNA repair and epigenetic changes. These data expand our knowledge of the molecular basis of diapause regulation and will be useful for future comparative approaches among annual killifish diapauses (I, II and III) and other organisms that exhibit dormancy. Further exploration of these proteins will help elucidate their possible functions during survival of dehydration stress due to aerial incubation during diapause.

## Materials and methods

### Collection and maintenance of adults

The protocols used in this work were approved by the Animal Experimentation Committee from the Universidad de la República, CHEA (Comisión Honoraria de Experimentación Animal; protocol code 240011-002308-14).

Adult females and males of *Austrolebias charrua* were collected in ephemeral ponds from La Coronilla, Departamento de Rocha, Uruguay (latitude _33.92, longitude _53.52). Adults were kept in 20-L tanks filled with reverse osmosis filtered water reconstituted with marine salt (Tetra Marine Salt Pro, 300 mS, pH 7–7.5) with continuous air bubbling. Fish were exposed to natural light, and the water temperature was maintained at 19°C. Water was partially changed every 5 days. Specimens were fed once a day with live bloodworms. Spawning occurred naturally with fish groups consisting of one male/two females isolated in tanks using a container filled with glass spheres (500 mm diameter Thomas Scientific, Swedesboro, NJ) on the bottom.

### Diapause III induction

Fertilized eggs were collected and kept in Yamamoto’s solution at 25°C [104] until the following features appeared: advanced organogenesis, pigmentation of the retina and skin and tip of the tail in contact with the head (stage 41, early prehatching [2, 105]). At this stage, the embryos were induced to DIII. To induce DIII, embryos were first rolled through filter paper to clean their surface. They were treated for 5 min with a 0.03% solution of sodium hypochlorite in Yamamoto’s solution to help prevent fungal and bacterial infections [18] and rinsed 2 times in Synergy water. Embryos were placed on wet filter paper with Yamamoto’s solution and kept in darkness in a plastic bag inside a container at room temperature. Humidity was checked periodically. Filter paper and bags were UV irradiated 45 min before use.

In terms of DIII embryos in natural environments, the only field data available come from the Mexican annual killifish *Millerichthys robustus* and *Nothobranchius* [29, 30]. Researchers found embryos arrested in DIII close to the end of the dry season before flood. Considering these data, to mimic these conditions, we placed the embryos in moistened paper in our experimental design.

After 3 months, embryos were observed by photonic microscopy with a blue filter (to minimize activation). DIII embryos were characterized by full development of the body with the tail extending over the head, most of the yolk consumed and cessation of mandibular and opercular contractions that mimic breathing in nondormant embryos (stage 43, late prehatching embryos [2, 105]).

We used three biological replicates per stage to compare differential protein expression profiles (Stage 41 early prehatching: 1 n = 20 embryos, 2n = 20 embryos, 3 n = 20 embryos; Stage 43 diapause III: 1 n = 17 embryos, n = 20 embryos, n = 8 embryos).

### Sample preparation for LC-MS/MS analysis

Protein extraction was performed following the protocol described by Link et al. [106] to avoid vitellogenin sample contamination.

Protein quantification was carried out by densitometry analysis following SDS-PAGE. For that purpose, samples were run in precast gels (NuPAGE™ 4 to 12%, Bis-Tris, 1.0 mm, Mini Protein Gel, 10-well, Invitrogen). The LMW-SDS Marker Kit (GE Healthcare) was used as a standard. After staining with colloidal Coomassie blue, gel images were digitized with a UMAX Power-Look 1120 scanner and LabScan 5.0 software (GE Healthcare). Quantification was performed using ImageQuant TL software (v8.1), a 1D analysis module.

Twenty micrograms of protein extracted from each sample was run on a 3-cm-long SDS-PAGE gel. Each lane was excised into 3 fragments, transferred to Eppendorf tubes and processed independently. Proteins were *in-gel* reduced and alkylated by incubation with 10 mM DTT for 1 h at 56°C followed by incubation with 50 mM iodoacetamide for 45 min at room temperature. *In-gel* protein digestion was performed by incubation with 2 μg of trypsin (sequence grade, Promega) in 50 mM ammonium bicarbonate overnight at 37°C. Peptides were extracted from gel pieces by the addition of 60% acetonitrile/0.1% formic acid with vigorous agitation followed by vacuum drying and resuspension in 0.1% formic acid. Samples were centrifuged at 16,000xg for 30 min at 4°C.

### LC-MS/MS analysis

Samples were analyzed by nano-LC MS/MS by means of a shotgun strategy using a nano-HPLC (EASY-nLC 1000, Thermo Scientific) coupled online to an LTQ Velos mass spectrometer (Thermo Scientific). Tryptic peptides were injected into an Acclaim® PepMap 100 nanotrap column (75 μm x 2 cm, Thermo Scientific) and separated on a 50 μm x 15 cm C18 Easy Spray column (PepMap® RSLC, 2 μM, 100 Å, Thermo Scientific) at a constant flow rate of 250 nL/min at 45°C. Peptide elution was achieved with a 70 min gradient from 0% to 55% mobile phase B (A: 0.1% formic acid; B: 0.1% formic acid in acetonitrile). Online MS analysis was carried out in data-dependent acquisition mode in two steps: acquisition of full MS scans in positive ion mode with an *m/z* range from 400 to 1200 Da followed by CID fragmentation of the ten most intense ions using a dynamic exclusion list (exclusion duration 45 s). The following parameters were set: spray voltage, 2.3 kV; capillary temperature, 260°C; normalized collision energy, 35; activation Q energy, 0.25; and activation time, 15 ms.

### Protein identification and analysis

PatternLab for Proteomics v4.0 software (http://www.patternlabforproteomics.org) was employed to generate a target-reverse database using sequences from the *Austrolebias charrua* genome [107]. Additionally, 127 common mass spectrometry contaminants were incorporated. Thermo raw files were searched against the database using the software integrated Comet search engine applying the following parameters: trypsin as proteolytic enzyme with full specificity and 2 missed cleavages allowed; oxidation of methionine as a variable modification, carbamido methylation of cysteines as a fixed modification; 800 ppm of tolerance from the measured precursor *m/z*. Each of the three fragmentation fractions belonging to the same sample were searched together, giving rise to a unique results file for each one. XCorr and Z-Score were used as the primary and secondary search engine scores, respectively. Peptide spectrum matches were filtered out using the Search Engine Processor (SEPro), and acceptable FDR criteria were set at 1% at the protein level. Comparison between proteins detected in each sample set was performed using PatternLab’s Approximately Area Proportional Venn Diagram module. Proteins uniquely detected in each stage were determined if they were found in at least two replicates of one condition and were absent from the other. PatternLab’s T-Fold module was used to detect proteins present in both conditions but at significantly different relative abundances by spectral count analysis.

The mass spectrometry proteomics data have been deposited to the ProteomeXchange Consortium via the PRIDE [108] partner repository with the dataset identifier PXD025196.

### Gene ontology and KEGG orthology analyses

GO terms were assigned using InterProScan v5.31–70.0 [109] with the option–goterms. GO enrichment was performed using the R package TopGO v2.34.0 [110], using all genes with an assigned GO (19.332 of 40.933 total proteins) as the universe and those genes either upregulated or exclusive to diapause III and prehatching as genes of interest. GO terms with a p-value <0.05 in the weighted Fisher test were considered significant. Enriched GO terms in each condition and their p-values were plotted in R (version 3.5.2, R Core Team, 2018 [111], using the ggplot2 (version 3.2.1, [112]) and cowplot (version 1.0.0, [113]) packages.

KEGG Orthology terms were assigned to all proteins using the KEGG Automatic Annotation Server [114] using GHOSTZ as the search algorithm and the SBH Method for assignment. The gene dataset included the default eukaryotic gene set plus the fish *Paramormyrops kingsleyae*, *Kryptolebias marmoratus (*mangrove rivulus*)*, *Takifugu rubripes (torafugu)*, *Sinocyclocheilus rhinocerous* and *Xiphophorus maculatus* (southern platyfish). KO terms were mapped to KEGG pathways using KEGG Mapper [115].

Protein sequences exclusively expressed or upregulated in each condition were analyzed for pathway enrichment using KOBAS3.0 [116] with default parameters (online version: http://kobas.cbi.pku.edu.cn/anno_iden.php), using *D*. *rerio* as a reference organism for analysis of KEGG pathways.

## Supporting information

S1 Table Overall list of identified proteins. (XLSX)

S2 Table List of proteins identified only in DIA, PRE, increased in DIA and increased in PRE. (XLSX)

S3 Table GO term. (XLSX)

S4 Table List of metabolic pathways detected by KEGG pathway analysis tool present in DIA and PRE conditions. (XLSX)

S5 Table List of proteins exclusively detected in DIA condition. (XLSX)

## References

[1] Lubzens E, Cerdà J, Clark M. Introduction. In Lubzens E, Cerdà J, Clark M, editors. Dormancy and Resistance in harsh environments. Curr. Genet. 21, Springer-Verlag, Berlin; 2010. pp 1–4.

[2] Wourms JP. The developmental biology of annual fishes I. Stages in the normal development of *Austrofundulus myersi* Dahl. J Exp Zool 1972a;182:143–168. 5079083 10.1002/jez.1401820202

[3] Podrabsky JE, Tingaud-Sequeira A, Cerda J. Metabolic dormancy and responses to environmental desiccation in fish embryos. In: Lubzens E Cerdà J, Clark M editors. Dormancy and Resistance in Harsh Environments, Vol 21. Curr. Genet. Springer-Verlag; 2010. pp 203–226.

[4] Hand SC, Denlnger DL, Podrabsky JE, Roy R. Mechanisms of annual diapause: recent developments from nematodes, crustaceans, insects and fish. Am J Phisiol Regul Integr Comp Physiol. 2016; 310: R1193–R1211. doi: 10.1152/ajpregu.00250.2015 27053646 PMC4935499

[5] Ragland GJ, Denlinger DL, Hahn DA. Mechanisms of suspended animation are revealed by transcript profiling of diapause in the flesh fly. Proc. Natl. Acad. Sci. U.S.A. 2010; 107: 14909–14914. doi: 10.1073/pnas.1007075107 20668242 PMC2930464

[6] Podrabsky JE, Hand SC. Physiological strategies during animal diapause: lessons from brine shrimp and annual killifish. J. Exp. Biol. 2015; 218: 1897–1906. doi: 10.1242/jeb.116194 26085666 PMC4487008

[7] Wourms JP. The developmental biology of fishes. III. Pre-embryonic diapause of variable duration in the eggs of annual fishes. J Exp Zool. 1972c; 182:389–414. doi: 10.1002/jez.1401820310 4674089

[8] Arezo MJ, D’Alessandro S, Papa N, de Sá R, Berois N. Sex differentiation pattern in the annual fish *Austrolebias charrua* (Cyprinodontiformes: Rivulidae). Tissue Cell. 2007; 39:89–98. doi: 10.1016/j.tice.2007.01.004 17399757

[9] Blazek R, Polacˇ ik M, Reichard M. Rapid growth, early maturationand short generation time in African annual fishes. Evo-Devo. 2013; 4:24. doi: 10.1186/2041-9139-4-24 24007640 PMC3844391

[10] Berois N, Arezo MJ, Papa NG, Clivio G. Annual fish: developmental adaptation for an extreme environment. Wire Dev Biol. 2012; doi: 10.1002/wdev.39 23801535

[11] Podrabsky JE, Riggs CL, Wagne JT. Tolerance of environmental stress. In: Berois N, García RO, de Sa R, editors. Annual fishes: life history strategy, diversity and evolution. Vol. 1 Boca Raton, FL: CRC Press Taylor and Francis. 2016a; pp 160–184.

[12] Furness AI. The evolution of an annual life cycle in killifish: adaptation to ephemeral aquatic environments through embryonic diapause. Biol Rev Camb Philos Soc. 2016; 91:796–812. doi: 10.1111/brv.12194 25969869

[13] Meller CL, Meller R, Simon RP, Culpepper KM, Podrabsky JE. Cell cycle arrest associated with anoxia-induced quiescence, anoxic preconditioning, and embryonic diapause in embryos of the annual killifish *Austrofundulus limnaeus*. J Comp Physiol B. 2012; 182:909–920. doi: 10.1007/s00360-012-0672-9 22570106 PMC3448833

[14] Woll SC, Podrabsky JE. Insulin-like growth factor signaling regulates developmental trajectory associated with diapause in embryos of the annual killifish *Austrofundulus limnaeus*. J Exp Biol. 2017; 220: 2777–2786. doi: 10.1242/jeb.151373 28515235

[15] Romney A, Podrabsky J. Transcriptomic analysis of maternally provisionedcues for phenotypic plasticity in the annual killifish, *Austrofundulus limnaeus*. EvoDevo. 2017; 8:6. doi: 10.1186/s13227-017-0069-7 28439397 PMC5401559

[16] Romney AL, Davis EM, Corona MM, Wagner JT, Podrabsky JE. Temperature-dependent vitamin D signaling regulates developmental trajectory associated with diapause in an annual killifish. Proc. Natl Acad Sci. 2018; 115, 12763–12768. doi: 10.1073/pnas.1804590115 30446615 PMC6294931

[17] Hu CK, Wang W, Brind’Amour J, Singh PP, Reeves GA, Lorincz MC, et al. Vertebrate diapause preserves organisms long term through Polycomb complex members. Science. 2020; 367: 870–874. doi: 10.1126/science.aaw2601 32079766 PMC7532943

[18] Podrabsky JE, Hand SC. The bioenergetics of embryonic diapause in an annual killifish, *Austrofundulus limnaeus*. J Exp Biol. 1999; 202:2567–2580. 10482717 10.1242/jeb.202.19.2567

[19] Levels PJ, Gubbels RE, Denuc e JM. Oxygen consumption during embryonic development of the annual fish *Nothobranchius korthausae* with special reference to diapause. Comp Biochem Physiol A Comp Physiol. 1986; 84:767–770. doi: 10.1016/0300-9629(86)90403-2 2875847

[20] Thompson AW, Ortí G. Annual killifish transcriptomics and candidate genes for metazoan diapause. Mol Biol Evol. 2016; 33:2391–5. doi: 10.1093/molbev/msw110 27297470

[21] Peters N. Embryonale Anpassungen oviparer Zahnkarpfen aus periodisch austrocknenden Gewassern. Int Rev Ges Hydrobiol 1963; 48:257–313.

[22] Murphy WJ, Collier GE. A molecular phylogeny for aplocheiloid fishes (Atherinomorpha, Cyprinodontiformes): the role of vicariance and the origins of annualism. Mol Biol Evol.1997; 14:790–799. doi: 10.1093/oxfordjournals.molbev.a025819 9254916

[23] Furness AI, Reznick DN, Springer MS, Meredith RW. Convergent evolution of alternative developmental trajectories associated with diapause in African and South American killifish. Proc R Soc Lond Series B Biol Sci. 2015; 282:1–9. doi: 10.1098/rspb.2014.2189 25631993 PMC4344141

[24] Loureiro M, de Sá RO, Serra W, Alonso F, Nielsen D, Calviño P, et al. Review of the family Rivulidae (Cyprinodontiformes, Aplocheiloidei) and a molecular and morphological phylogeny of the annual fish genus *Austrolebias* Costa 1998. Neotrop Ichthyol. 2018; 16(3): e180007. doi: 10.1590/1982-0224-20180007

[25] Probides. Plan director. Reserva de Biosfera “Bañados del Este”. 1999; p 159. Rocha, Uruguay.

[26] Hu C, Brunet A. The African turquoise killifish: A research organism to study vertebrate aging and diapause. Aging Cell. 2018; e12757. doi: 10.1111/acel.12757 29573324 PMC5946070

[27] Reichard M, Polačik M. The Natural History of Model Organisms: *Nothobranchius furzeri*, an ‘instant’ fish from an ephemeral habitat. 2019. Elife 8, e41548. doi: 10.7554/eLife.41548 PMC6324871 30616713

[28] Ziv T, Chalifa-Caspi V, Denekamp N, Plaschkes I, Kierszniowska S, Blais I, et al. Dormancy in embryos: insight from hydrated encysted embryos of an aquatic invertebrate. Mol Cell Proteomics. 2017; 16:1746–1769. doi: 10.1074/mcp.RA117.000109 28729386 PMC5629262

[29] Domínguez-Castanedo O, Valdesalici S, Rosales-Torres AM. Developmental ecology of annual killifish *Millerichthys robustus* (Cyprinodontiformes: Cynolebiidae). Dev Dyn. 2017; 246:802–806. doi: 10.1002/dvdy.24519 28493325

[30] Polačik M, Vrtílek M, Reichard M, Žák J, Blažek R, Podrabsky J. Embryo ecology: Developmental synchrony and asynchrony in the embryonic development of wild annual fish populations. EcolEvol. 2021;00: 1–12. doi: 10.1002/ece3.7402 33976861 PMC8093744

[31] Schiesari L, O’Connor MB. Diapause. Delaying the developmental clock in response to a changing environment. Curr. Topics Dev. Biol. 2013; 105: 213–246. doi: 10.1016/B978-0-12-396968-2.00008-7 23962844

[32] Zajic DE, Podrabsky JE. Oxidative stress and its effects during dehydration. Comp Biochem Physiol A Mol Integr Physiol. 2020; 146: 621–631. 10.1016/j.cbpa.2006.02.030 16580854

[33] Malik AI, Storey KB. Activation of antioxidant defense during dehydration stress in the African clawed frog. Gene. 2009; 442: 99–107. doi: 10.1016/j.gene.2009.04.007 19379800

[34] Gary N, Landis JT. Superoxide dismutase evolution and life span regulation. Mech Ageing Dev. 2005; 126:365–79). doi: 10.1016/j.mad.2004.08.012 15664623

[35] Wagner JT, Knapp MJ, Podrabsky J. Antioxidant capacity and anoxia-tolerance in *Austrofundulus limnaeus* embryo. J Exp Biol. 2019; 222: 12 jeb204347. 10.1242/jeb.204347 31160427

[36] Sim C, Denlinger DL. Catalase and superoxide dismutase-2 enhance survival and protect ovaries during overwintering diapause in the mosquito *Culex pipiens*. J Insect Physiol. 2011;57:628–634. doi: 10.1016/j.jinsphys.2011.01.012 21277308 PMC3104096

[37] Sim C, Denlinger DL. Insulin signaling and FOXO regulate the overwintering diapause of the mosquito *Culex pipiens*. Proc Natl Acad Sci U S A. 2008; 105:6777–6781. doi: 10.1073/pnas.0802067105 18448677 PMC2373331

[38] Klotz LO, Sánchez-Ramos C, Prieto-Arroyo I, Urbánek P, Steinbrenner H, Monsalve M. Redox regulation of FoxO transcription factors. Redox Biol 2015; 6: 51–72. doi: 10.1016/j.redox.2015.06.019 26184557 PMC4511623

[39] Lant B, Storey KB. An overview of stress response and hypometabolic strategies in *Caenorhabditis elegans*: conserved and contrasting signals with the mammalian system. Int J Biol Sci. 2010; 6:9–50. doi: 10.7150/ijbs.6.9 20087441 PMC2808051

[40] Singh S, Brocker C, Koppaka V, Chen Y, Jackson BC, Matsumoto A, et al. Aldehyde dehydrogenases in cellular responses to oxidative/electrophilic stress. Free Radic Biol Med. 2013; 56: 89–101. doi: 10.1016/j.freeradbiomed.2012.11.010 23195683 PMC3631350

[41] Jin L, Li D, Alesi GN, Fan J, Kang HB, Lu Z, et al. Glutamate dehydrogenase 1 signals through antioxidant glutathione peroxidase 1 to regulate redox homeostasis and tumor growth. Cancer Cell. 2015; 27:257–70. doi: 10.1016/j.ccell.2014.12.006 25670081 PMC4325424

[42] Fan J, Teng X, Liu L, Mattaini K, R, Looper R E, Vander Heiden M G, et al. Human phosphoglycerate dehydrogenase produces the oncometabolite D-2-hydroxyglutarate. ACS Chem. Biol. 2015;10 510–516. doi: 10.1021/cb500683c 25406093 PMC4340346

[43] Du X, Hu H. The Roles of 2-Hydroxyglutarate. Front. Cell Dev. Biol. 2021; 9, 651317. doi: 10.3389/fcell.2021.651317 33842477 PMC8033037

[44] Zajic DE, Podrabsky JE. Metabolomics analysis of annual killifish (*Austrofundulus limnaeus*) embryos during aerial dehydration stress. Physiol Genomics. 2020; 52:408–42. doi: 10.1152/physiolgenomics.00072.2020 32776802

[45] Lee P, Chandel NS, Simon MC. Cellular adaptation to hypoxia through hypoxia inducible factors and beyond. Nat Rev Mol Cell Biol. 2020; 21:268–283. doi: 10.1038/s41580-020-0227-y 32144406 PMC7222024

[46] Rinehart JP, Li A, Yocum GD, Robich RM, Hayward SA, Denlinger DL. Up-regulation of heat shock proteins is essential for cold survival during insect diapause. Proc Natl Acad Sci U S A. 2007;104:11130–11137. doi: 10.1073/pnas.0703538104 17522254 PMC2040864

[47] Saravanakumar R, Ponnuvel KM, Qadri SMH. Expression of metabolic enzyme genes and heat-shock protein genes during embryonic development in diapause and non-diapause egg of multivoltine silkworm *Bombyx mori*. Biologia 2008; 63:737–744.

[48] Wolschin F, Gadau J. Deciphering proteomic signatures of early diapause in *Nasonia*. PloS One. 2009; 4:7 e6394. 28 doi: 10.1371/journal.pone.0006394 19636376 PMC2712079

[49] Hayward SA, Pavlides SC, Tammariello SP, Rinehart JP, Denlinger DL. Temporal expression patterns of diapause-associated genes in flesh fly pupae from the onset of diapause through post-diapause quiescence. J Insect Physiol. 2005; 51:631–40. doi: 10.1016/j.jinsphys.2004.11.009 15993127

[50] King AM, MacRae TH. Insect heat shock proteins during stress and diapause. Annu Rev Entomol. 2015; 7:59–75. doi: 10.1146/annurev-ento-011613-162107 25341107

[51] Li AQ, Popova-Butler A, Dean DH, Denlinger DL. Proteomics of the flesh fly brain reveals an abundance of upregulated heat shock proteins during pupal diapause. J insect Physiol. 2007; 53: 385–391. doi: 10.1016/j.jinsphys.2007.01.003 17349654

[52] Carter CA, Wourms JP. An Ultrastructural Analysis of the Dispersed Cell Phase During Development of the Annual Fish, *Cynolebias*. J Morphol. 1990; 204: 209–225. doi: 10.1002/jmor.1052040209 29865708

[53] Wilson JE. Isozymes of mammalian hexokinase: structure, subcellular localization and metabolic function. J Exp Biol. 2003 Jun; 206(Pt 12):2049–57. doi: 10.1242/jeb.00241 12756287

[54] Rosa JC, César MC. Role of Hexokinase and VDAC in Neurological Disorders. Curr Mol Pharmacol. 2016;9(4):320–331. doi: 10.2174/1874467209666160112123036 26758954

[55] Shoshan-Barmatz V, Zakar M, Rosenthal K, Abu-Hamad S. Key regions of VDAC1 functioning in apoptosis induction and regulation by hexokinase. Biochim Biophys Acta. 2009; 1787(5):421–30. doi: 10.1016/j.bbabio.2008.11.009 19094960

[56] Shoshan-Barmatz V, Mizrachi D. VDAC1: from structure to cancer therapy. Front Oncol. 2012; 2. doi: 10.3389/fonc.2012.00002 23233904 PMC3516065

[57] Hussein AM, Wang Y, Mathieu J, Margaretha L, Song C, Jones DC, et al. Metabolic Control over mTOR-Dependent Diapause-like State. Dev Cell. 2020; 52:236–250. doi: 10.1016/j.devcel.2019.12.018 31991105 PMC7204393

[58] Yaginuma T, Yamashita O. Polyol metabolism related to diapause in Bombyx eggs: Different behaviour of sorbitol from glycerol during diapause and post-diapause. J Insect Physiol. 1978; 24:347–354.

[59] Ahlquist DA, Nelson RA, Steiger DL, Jones JD, Ellefson RD. Glycerol metabolism in the hibernating black bear. J Comp Physiol B. 1984; 155:75–79.

[60] Chino H. Carbohydrate metabolism in the diapause egg of the silkworm, *Bombyx mori*—II: Conversion of glycogen into sorbitol and glycerol during diapause Journal of Insect Physiology. 1958; 2:1–4, IN1, 5–12.

[61] Rusu P, Shao C, Neuerburg A, Acikgöz AA, Wu Y, Zou P, et al. GPD1 Specifically Marks Dormant Glioma Stem Cells with a Distinct Metabolic Profile. Cell Stem Cell. 2019; 25:241–257.e8. doi: 10.1016/j.stem.2019.06.004 31303549

[62] Albertyn J Hohmann S, Thevelein JM, Prior BA. GPD1, which encodes glycerol-3-phosphate dehydrogenase, is essential for growth under osmotic stress in *Saccharomyces cerevisiae*, and its expression is regulated by the high-osmolarity glycerol response pathway. Mol Cell Biol. 1994; 14:4135–44. doi: 10.1128/mcb.14.6.4135-4144.1994 8196651 PMC358779

[63] Zhao Y, Li X, Wang F, Zhao X, Gao Y, Zhao C, et al. Glycerol-3-phosphate dehydrogenase (GPDH) gene family in Zea mays L.: Identification, subcellular localization, and transcriptional responses to abiotic stresses. PLoS ONE. 2018; 13: e0200357. doi: 10.1371/journal.pone.0200357 29990328 PMC6039019

[64] Hahn DA, Denlinger DL. Energetics of Insect Diapause Annu Rev Entomol. 2011; 56:103–121. doi: 10.1146/annurev-ento-112408-085436 20690828

[65] Arrese EL, Soulages JL. Insect fat body: energy, metabolism, and regulation. Annu Rev Entomol. 2010; 55:207–225. doi: 10.1146/annurev-ento-112408-085356 19725772 PMC3075550

[66] Liu F, Xiao Y, Ji XL, Zhang KQ, Zou CG. The cAMP-PKA pathway-mediated fat mobilization is required for cold tolerance in *C*. *elegans*. Sci Rep. 2017; 7: 638. doi: 10.1038/s41598-017-00630-w 28377576 PMC5428847

[67] Kim YM, Barak LS, Caron MG, Benovic JL. Regulation of arrestin-3 phosphorylation by casein kinase II. J Biol Chem. 2002 May 10; 277:16837–46. doi: 10.1074/jbc.M201379200 11877451

[68] Mattera R, Yatani A, Kirsch GE, Graf R, Okabe K, Olate J, et al. Recombinant alpha i-3 subunit of G protein activates Gk-gated K+ channels. J Biol Chem. 1989;264:465–71. 2535845

[69] Yocu GD. Environmental regulation of the purine synthesis enzyme purH transcript during adult diapause in *Leptinotarsa decemlineata* (Coleoptera: Chrysomelidae). Eur J Entomol. 2004;101:199–203.

[70] Snider NT, Omary MB. Post-translational modifications of intermediate filament proteins: mechanisms and functions. Nat Rev Mol Cell Biol. 2014; 15:163–177. doi: 10.1038/nrm3753 24556839 PMC4079540

[71] Moll R, Divo M, Langbein L The human keratins: biology and pathology. Histochem Cell Biol. 2008; 129:705–733. doi: 10.1007/s00418-008-0435-6 18461349 PMC2386534

[72] Tao GZ, Looi KS, Toivola DM, Strnad P, Zhou Q, Liao J, et al. Keratins modulate the shape and function of hepatocyte mitochondria: a mechanism for protection from apoptosis. J Cell Sci. 2009; 122:3851–3855. doi: 10.1242/jcs.051862 19825937 PMC2773188

[73] Bozza WP, Zhang Y, Zhang B. Cytokeratin 8/18 protects breast cancer cell lines from TRAIL-induced apoptosis. Oncotarget. 2018; 9:23264–23273. doi: 10.18632/oncotarget.25297 29796187 PMC5955420

[74] Nurdan G, Valentyn U, Kateryna L, Christian T, Marianne Z, Pierre N, et al. Keratins 8 and 18 are type II acute-phase responsive genes overexpressed in human liver disease. Liver Int. 2015; 35:1203–1212. doi: 10.1111/liv.12608 24930437

[75] Djudjaj S, Papasotiriou M, Bülow RD, Wagnerova A, Lindenmeyer MT, Cohen CD, et al. Keratins are novel markers of renal epithelial cell injury. Kidney Int. 2016; 89:792–808. doi: 10.1016/j.kint.2015.10.015 26924053

[76] Zupancic T, Stojan J, Lane EB, Komel R, Bedina-Zavec A, Liovic M Intestinal cell barrier function in vitro is severely compromised by keratin 8 and 18 mutations identified in patients with inflammatory bowel disease. PLoS ONE. 2014; 9:e99398. doi: 10.1371/journal.pone.0099398 24915158 PMC4051775

[77] Tiwari R, Sahu I, Soni BL, Sathe GJ, Thapa P, Patel P, et al. Depletion of keratin 8/18 modulate oncogenic potential by governing multiple signaling pathways. FEBS J. 2018; 285:1251–1276. doi: 10.1111/febs.14401 29427328

[78] Ku NO, Michie S, Resurreccion EZ, Broome RL, Omary MB. Keratin binding to 14‑3‑3 proteins modulates keratin filaments and hepatocyte mitotic progression. Proc. Natl Acad. Sci. USA. 2002; 99:4373–4378. doi: 10.1073/pnas.072624299 11917136 PMC123655

[79] Pennington KL, Chan TY, Torres MP, Andersen JL. The dynamic and stress‐adaptive signaling hub of 14‐3‐3: Emerging mechanisms of regulation and context‐dependent protein‐protein interactions. Oncogene. 2018; 37:5587–5604. doi: 10.1038/s41388-018-0348-3 29915393 PMC6193947

[80] Mor-Vaknin N, Punturieri A, Sitwala K, Markovitz DM. Vimentin is secreted byactivated macrophages, Nat Cell Biol. 2003; 5: 59–63. doi: 10.1038/ncb898 12483219

[81] Frescas D, Roux CM, Aygun-Sunar S, Gleiberman AS, Krasnov P, Kurnasov OV, et al. Senescent cells expose and secrete an oxidized form of membrane-bound vimentinas revealed by a natural polyreactive antibody, Proc Natl Acad Sci U S A. 2017;114:E1668–E1677. doi: 10.1073/pnas.1614661114 28193858 PMC5338544

[82] Ivaska J, Pallari HM, Nevo J, Eriksson, JE. Novel functions of vimentin in cell adhesion, migration, and signaling, Exp Cell Res. 2007; 313:2050–62. doi: 10.1016/j.yexcr.2007.03.040 17512929

[83] Chang L, Goldman RD. Intermediate filaments mediate cytoskeletal crosstalk. Nat Rev Mol Cell Biol. 2004; 5:601–13. doi: 10.1038/nrm1438 15366704

[84] Morrow CS, Porter TJ, Xu N, Arndt ZP, Ako-Asare K, Heo HJ, et al. Vimentin coordinates protein turnover at the aggresome during neural stem cell quiescence exit Cell Stem Cell. 2020; 26:2. 558–568.e9. doi: 10.1016/j.stem.2020.01.018 Epub 2020 27 32109376 PMC7127969

[85] Ruhrberg C, Hajibagheri MA, Parry DA, Watt FM. Peri-plakin, a novel component of cornified envelopes and desmosomes that belongs to the plakin family and forms complexes with envoplakin. J. Cell Biol. 1997;139:1835–1849. doi: 10.1083/jcb.139.7.1835 9412476 PMC2132639

[86] Heuvel APJ van den. Analysis of the protein kinase B-Forkhead box O signaling pathway. PhD Thesis, Utrecht University. 2004. Available from: dspace.library.uu.nl/handle/1874/1072.

[87] Lam EW, Brosens JJ, Gomes AR, Koo CY. Forkhead box proteins: Tuning forks for transcriptional harmony. Nat. Rev. Cancer. 2013;13:482–495. doi: 10.1038/nrc3539 23792361

[88] Bowman CJ, Ayer DE, Dynlacht BD. Foxk proteins repress the initiation of starvation-induced atrophy and autophagy programs. Nat Cell Biol. 2014; 16:1202–14. doi: 10.1038/ncb3062 25402684 PMC4250422

[89] Sakaguchi M, Cai W, Wang CH, Cederquist CT, Damasio M., Homan EP, et al. FoxK1 and FoxK2 in insulin regulation of cellular and mitochondrial metabolism Nat. Commun. 2019; 10: 1582. doi: 10.1038/s41467-019-09418-082 PMC6450906 30952843

[90] Amaya MJ, Oliveira AG, Guimarães ES, Casteluber MCF, Carvalho SM, Andrade LM, et al. The insulin receptor translocates to the nucleus to regulate cell proliferation in liver. Hepatology. 2014; 59:274–283. doi: 10.1002/hep.26609 23839970 PMC3823683

[91] Wang W, Li X, Lee M, Jun S, Aziz KE, Feng L, et al. FOXKs promote Wnt/β-catenin signaling by translocating DVL into the nucleus. Dev Cell. 2015; 32: 707–718. doi: 10.1016/j.devcel.2015.01.031 25805136 PMC4374128

[92] Lee DC, Kang YK, Kim WH, Jang YJ, Kim DJ, Park IY, et al. Functional and clinical evidence for NDRG2 as a candidate suppressor of liver cancer metastasis. Cancer Res. 2008; 68:4210–4220. doi: 10.1158/0008-5472.CAN-07-5040 18519680

[93] Kim YJ, Yoon SY, Kim JT, Song EY, Lee HG, Son HJ, et al. NDRG2 expression decreases with tumor stages and regulates TCF/beta-catenin signaling in human colon carcinoma, Carcinogenesis. 2009; 30:598–605. doi: 10.1093/carcin/bgp047 19237607 PMC2664458

[94] Park Y, Shon SK, Kim A, Kim K, Yang Y, Cho DH, et al. SOCS1 induced by NDRG2 expression negatively regulates STAT3 activation in breast cancer cells, Biochem. Biophys. Res. Commun. 2007; 363:361–367. doi: 10.1016/j.bbrc.2007.08.195 17888401

[95] Ferry RJ Jr, Katz LE, Grimberg A, Cohen P, Weinzimer SA. Cellular actions of insulin-like growth factor binding proteins. Horm Metab Res. 1999; 31:192–202. doi: 10.1055/s-2007-978719 10226802 PMC4151550

[96] Clemmons DR, Snyder DK, Busby WH Jr. Variables controlling the secretion of insulin-like growth factor binding protein-2 in normal human subjects. J Clin Endocrinol Metab. 1991; 73:727–733. doi: 10.1210/jcem-73-4-727 1716260

[97] Sancar A. Structure and function of DNA photolyase and cryptochrome bluelight photoreceptors. Chem Rev. 2003; 103:2203–2237. doi: 10.1021/cr0204348 12797829

[98] Wagner JT, Podrabsky JE. Extreme tolerance and developmental buffering of UV-C induced DNA damage in embryos of the annual killifish *Austrofundulus limnaeus*. J Exp Zool. 2015; 323A:10–30. doi: 10.1002/jez.1890 25387429

[99] Parkhitko AA, Jouandin P, Mohr SE, Perrimon N. Methionine metabolism and methyltransferases in the regulation of aging and lifespan extension across species. Aging Cell. 2019; 18: e13034. doi: 10.1111/acel.13034 31460700 PMC6826121

[100] Yang H, Berry S, Olsson TSG, Hartley M, Howard M, Dean C. Distinct phases of Polycomb silencing to hold epigenetic memory of cold in *Arabidopsis*. Science. 2017; 357:1142–1145. doi: 10.1126/science.aan1121 28818969

[101] Reynolds JA. Epigenetic Influences on Diapause. Adv In Insect Phys. 2017; 53:115–143.

[102] Levels PJ, Denuce JM. Intrinsic variability in the frequency of embryonic diapauses of the annual fish *Nothobranchius korthausae*, regulated by light:dark cycle and temperature. Environ Biol Fishes. 1988; 22:211–223. doi: 10.1007/BF00005382

[103] Podrabsky JE, Romney AL, Culpepper KM. Alternate developmental pathway. In: Berois N, García RO, de Sa R, editors. Annual fishes: life history strategy, diversity and evolution. Vol. 1 Boca Raton, FL: CRC Press Taylor and Francis. 2016b. pp. 63–73.

[104] Yamamoto T. Medaka. In: Wilt FH, Wessels N, editors. Methods in developmental biology. New York: Thomas and Crowell Company. 1967. pp. 101–111.

[105] Podrabsky JE, Riggs CL, Romney AL, Woll SC, Wagner JT, Culpepper KM, et al. Embryonic development of the annual killifish *Austrofundulus limnaeus*: An emerging model for ecological and evolutionary developmental biology research and instruction. Dev Dyn. 2017; 246: 779–801. doi: 10.1002/dvdy.24513 28481428

[106] Link V, Shevchenko A, Heisenberg CP. Proteomics of early zebrafish embryos. BMC Dev Biol. 2006; 6:1. doi: 10.1186/1471-213X-6-1 16412219 PMC1363346

[107] Gajardo F, Di Genova A, Valdivieso C, Pereiro L, Arezo MJ, Nardocci G, et al. Accelerated genome expansion in an annual South American killifish. Manuscript in preparation.

[108] Perez-Riverol Y, Csordas A, Bai J, Bernal-Llinares M, Hewapathirana S, Kundu DJ, et al. The PRIDE database and related tools and resources in 2019: improving support for quantification data. Nucleic Acids Res. 2019; 47(D1):D442–D450 (PubMed ID: 30395289). doi: 10.1093/nar/gky1106 30395289 PMC6323896

[109] Jones P, Binns D, Chang HY, Fraser M, Li W, McAnulla C, et al. InterProScan 5: genome-scale protein function classification. Bioinformatics. 2014. 30:1236–1240. doi: 10.1093/bioinformatics/btu031 24451626 PMC3998142

[110] Alexa A, Rahnenfuhrer J. topGO: Enrichment Analysis for Gene Ontology. 2016; Available from:https://bioconductor.riken.jp/packages/3.4/bioc/html/topGO.html

[111] R Core Team. R: A Language and Environment for Statistical Computing. R Foundation for Statistical Computing, Vienna, Austria. 2018; https://www.R-project.org

[112] Wickham H. ggplot2: Elegant Graphics for Data Analysis. Springer-Verlag New York. 2016.

[113] Wilke CO. cowplot: Streamlined Plot Theme and Plot Annotations for “ggplot2.” 2019.

[114] Moriya Y, Itoh M, Okuda S, Yoshizawa AC, Kanehisa M. KAAS: an automatic genome annotation and pathway reconstruction server. Nucleic Acids Res. 2007; 35, W182–W185. doi: 10.1093/nar/gkm321 17526522 PMC1933193

[115] Kanehisa M, Sato Y. KEGG Mapper for inferring cellular functions from protein sequences. Protein Sci. Publ. Protein Soc. 2019; doi: 10.1002/pro.3711 31423653 PMC6933857

[116] Xie C, Mao X, Huang J, Ding Y, Wu J, Dong S, et al. KOBAS 2.0: a web server for annotation and identification of enriched pathways and diseases. Nucleic Acids Res. 2011; 39, W316–322. doi: 10.1093/nar/gkr483 21715386 PMC3125809

